# USP10 promotes the progression and attenuates gemcitabine chemotherapy sensitivity via stabilizing PLK1 in PDAC

**DOI:** 10.1038/s41419-025-07757-z

**Published:** 2025-06-14

**Authors:** Xuezhi Du, Runze Yu, Caigu Yan, Penggang Dong, Cheng Wei, Bo Wang, Chenhui Zhang, Yingjie He, Yaqing Wei, Lei Han, Jinjin Sun

**Affiliations:** 1 Department of Hepatopancreatobiliary Surgery, The Second Hospital of Tianjin Medical University, Tianjin, China; 2 Key Laboratory of Post‑Neuroinjury Neuro‑Repair and Regeneration in Central Nervous System, Ministry of Education and Tianjin City, Tianjin Neurological Institute, Tianjin Medical University General Hospital, Tianjin, China; 3 Department of Colorectal Surgery Ward I, Shandong Cancer Hospital and Institute, Shandong First Medical University and Shandong Academy of Medical Sciences, Jinan, Shandong China; 4 Department of Hepatobiliary Surgery, Changzhi People’s Hospital, Affiliated to Changzhi Medical College, Changzhi, Shanxi China

**Keywords:** Oncogenes, Cell biology, Gastrointestinal cancer

## Abstract

Pancreatic ductal adenocarcinoma (PDAC) is one of the most malignant tumors with limited treatment options, and chemotherapy resistance contributes to poor prognosis. An increasing number of studies have shown that ubiquitin specific peptidases (USPs), a subtype of deubiquitinases, can affect tumor progression by regulating the stability or biological function of substrate proteins. Thus, USPs are becoming attractive targets for cancer treatment. In this study, we investigated the role of USPs in PDAC. This study illustrated significant upregulation of USP10 expression in PDAC, which was found to be correlated with unfavorable prognosis. Further evaluation showed that USP10 exhibited the ability to facilitate PDAC progression in vitro and in vivo. The assays of immunoprecipitation-mass spectrometry, CO-IP, and GST pull-down suggested that USP10 directly interacted with PLK1. Deubiquitination assays indicated that USP10 could reduce the ubiquitination of PLK1 and increase protein stability. Moreover, USP10 may promote autophagy in PDAC cells through PLK1 and further attenuate the response of PDAC cells to gemcitabine (GEM). Finally, we demonstrated that the inhibition of USP10 combined with GEM synergistically inhibited the progression of PDAC in vitro and in vivo. In summary, we revealed that USP10, as a tumor promoter, promoted the progression and attenuated GEM chemotherapy sensitivity via stabilizing PLK1 in PDAC, providing a potential target for the treatment of PDAC.

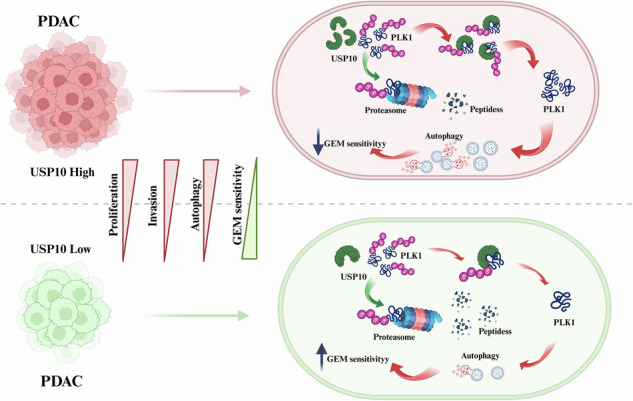

## Introduction

Pancreatic cancer (PC) is among the most common malignancies worldwide [1]. The ranking of PC mortality increased from seventh in 2020 to sixth in 2022 [2, 3]. The most common type of PC is pancreatic ductal adenocarcinoma (PDAC) [4]. Difficulty in early diagnosis and resistance to chemotherapy poses substantial challenges in the clinical treatment of PDAC. Radical surgery and chemotherapy are the first-line treatments for PDAC [5]. On the other hand, targeted therapy and immunotherapy have developed slowly for PDAC, and only a small number of PDAC patients benefit from targeted therapy and immunotherapy because of the limited targets and immunosuppressive microenvironment [6, 7]. Despite great efforts in PDAC treatment, the current 5-year survival rate is only 12% [8]. Thus, further exploration of the possible therapeutic options to improve patient prognosis is urgently needed.

Ubiquitination is a classical posttranslational modification of proteins [9]. Several studies have suggested that ubiquitination contributes to the progression of different cancers [10–12]. The level of substrate protein ubiquitination is regulated by both ubiquitin ligases and deubiquitinases (DUBs), which are dynamic and reversible processes [13]. Ubiquitin-specific peptidase 10 (USP10), a DUB, plays a complex role in tumor progression and can act as both a tumor promoter and suppressor in different cancers [14]. The present studies suggest that USP10 can promote the progression of PDAC [15, 16]. Thus, USP10 is an attractive therapeutic target, and is worth exploring the molecular mechanism of its function in PDAC. PLK1 is a conserved Ser/Thr kinase that regulates the cell cycle. Moreover, PLK1 acts as a tumor promoter in a variety of cancers [17] and modulates autophagy [18]. Although protein stability of PLK1 can be regulated by DUBs in different cancers [19, 20], the deubiquitination network of PLK1 in PDAC needs to be further defined.

Gemcitabine (GEM) has improved the prognosis of advanced PC as a first-line chemotherapeutic agent since 1997 [21]. Nevertheless, the prognosis of PDAC patients remains suboptimal owing to resistance to GEM chemotherapy [22]. There are many factors that influence the sensitivity of PC to GEM, such as autophagy, hypoxia, glycolysis, etc. [23]. GEM can induce autophagy in PC cells [24], which is a significant reason for the resistance of PDAC to GEM chemotherapy [25]. These studies revealed the close interactions between GEM chemotherapy resistance and autophagy. Thus, targeting autophagy is a promising method for regulating chemotherapy resistance [26]. Currently, the role of USP10 in GEM sensitivity and autophagy in PDAC remains unclear and requires further investigation.

Our study showed that USP10 was upregulated and associated with an unfavorable prognosis in PDAC. USP10 promoted the progress of PDAC in vitro and in vivo. Moreover, we demonstrated that USP10 interacted with PLK1 and increased protein stability by deubiquitinating PLK1. Furthermore, USP10 promoted autophagy through PLK1, thus attenuated GEM chemotherapy sensitivity in PDAC. Finally, our study demonstrated that the inhibition of USP10 combined with GEM synergistically inhibited the progression of PDAC in vitro and in vivo, providing a potential treatment for PDAC.

## Methods

### Data acquisition and processing

The PDAC data of TCGA and CPTAC were downloaded from cBioPortal database (https://www.cbioportal.org/). The PDAC data of GSE62452, GSE28735, GSE43797 were obtained from GEO database (https://www.ncbi.nlm.nih.gov/geo/). Limma package was used to obtain the differentially expressed USP family members (USPs). Survival package was used to perform survival analysis, univariate and multivariate Cox regression analysis. The correlation tables between USP10 and other genes were obtained from LinkedOmics database (https://www.linkedomics.org), and USP10-related genes were identified according to the criterion of |R| > 0.3, *P* < 0.05.

### Immunohistochemistry (IHC)

The paraffin-embedded PDAC specimens of 18 patients were obtained from the Second Hospital of Tianjin Medical University from January 2019 to December 2023. This study was performed according to the principles of the Declaration of Helsinki. This study was approved by the Human Scientific Ethics Committee of the Second Hospital of Tianjin Medical University, and the informed consent was written and provided by patients after being fully informed. The method of IHC was described previously [27]. The IHC assay was performed based on the instructions of the IHC kit (PV-9000, ZSGB-BIO, China). In brief, 5μm slides were roasted at 60 °C for 2 h, and then treated in xylene, alcohol, and ddH_2_O. Then the slides were heated in sodium citrate solution at 98 °C for 15 min and washed with PBS for three times. After incubation with buffer 1 for 15 min, the slides underwent a 30-min incubation at 37 °C in the presence of a 5% BSA solution. The slides were incubated with antibody of USP10 (19374-1-AP, proteintech, China) diluted at 1:100 at 4 °C for 8 h. Then the slides were incubated with buffer 2 for 20 min and washed by PBS and incubated with buffer 3 for 30 min at 37 °C. Then the slides were stained by DAB for 10 min and treated with Hematoxylin (G1120, Solarbio, China). At last, the slides were dehydrated and mounted. And the Olympus BX53 up-right microscope was used to collected the data. The IHC score was calculated based on the method described by previous study [28].

### Cell culture

The method of cell culture was described previously [29]. In brief, PANC-1, MIAPaCa-2, AsPC-1, SW1990, and HEK 293 were purchased from cell bank of Chinese Academy of Sciences. Mycoplasma testing and STR sequencing were implemented. PANC-1, MIAPaCa-2, and HEK 293 were cultured in DMEM (Corning, USA) medium containing 10% fetal bovine serum (FBS, 04-001-1 A, Bioind, Israel). RPMI-1640 (Gibco, USA) medium was used to culture AsPC-1. All cells were cultured in 37 °C constant temperature incubator containing 5% CO_2_. All cell lines were identified by STR to exclude contamination from other cell lines and were determined to be free of mycoplasma.

### RNA extraction and RT-qPCR

The cells were lysed by RNAiso Plus (9108, Takara, Japan), and the total RNA was extracted. The process of reverse transcription was performed as reagent instructions (11141ES, Yeasen, China). For qPCR, 2 μl primer and 2 μl cDNA were added into each tube, and configured into 20 μl qPCR system according to qPCR regent instructions (11184ES, Yeasen, China). The process of qPCR was performed as previous study, described [30]. The corresponding CT values were recorded and compared quantitatively by 2^−△△CT^ method. The primer sequences were shown in Supplementary Table 1.

### siRNA, plasmids, transfection, and lentivirus infection

The siRNA targeting USP10 and PLK1 were purchased from GenePharma Co., Ltd, and the sequences were provided in supplementary Table 2. All plasmids were supplied by GenScript Co., Ltd, and detailed information was shown in Supplementary Table 3. The siRNA, plasmids, shUSP10, and lentivirus of Mcherry-EGFP-LC3B were transfected into cells as previous study, described [31]. The sequence of shUSP10 is shown as follows: sense 5′-CCAUAAAGAUU GCAGAGUUTT-3′; antisense 5′-AACUCUGCAAUCUUUAUGGTT-3′.

### Western blotting

The process of western blotting was performed as previous study, described [32]. And the information of antibodies was shown in Supplementary Table 4.

### Cell proliferation assays

The CCK8 assay was performed based on the regent instructions (HY-K0301, MCE, USA). The EdU kit was provided by Ribobio Co., Ltd (C10310-3, Guangzhou, China), and EdU assay was performed based on the instructions. For colony formation assay, 1 × 10^3^ cells were put into 6 well plate and change the medium every 3 days until it had been cultured for 2 weeks. At last, clones containing more than 50 cells were counted.

### Cell migration and invasion assays

The cells were treated with 5 μg/mL Mitomycin C for 12 h before the assays. For wound healing assay, 2 × 10^5^ cells were put into 6-well plate. Once the cells adhered to the surface, a scratch was created using a 200 μl pipette tip and cultured with FBS-free medium. The images were collected at 0 h, 12 h, 24 h. For Transwell assay, Matrigel matrix glue (356234, Corning, USA) was used. 2 × 10^4^ cells resuspended in 400 μl FBS-free medium were put into upper chamber, 600 μl total medium was put into lower chamber. After 24 h, the cells were fixed and stained, and the data were collected by microscope (BX53, Olympus, Japan).

### Animal studies

The animal studies were approved by the Animal Ethical and Welfare Committee of the Second Hospital of Tianjin Medical University. Male BALB/c nude mice of 4 weeks were provided by Gempharmatech Co. (Jiangsu, China). The PANC-1 cells were used to construct the subcutaneous xenograft model. The siRNA solution was injected into the tumor once the tumor size reached 100mm^3^. The siRNA solution was composed of siRNA and lipofectamine^TM^ 3000 in a volume ratio of 1:1. Each nude mouse received an injection of 20 μl siRNA solution. The volume was calculated as shown below: V = (length×width^2^)/2. After 30 days, the tumors were harvested, and the mass of the tumors was weighed. When the synergistic effects of USP10 knockdown and GEM were explored in vivo, the PANC-1 cells were infected with the lentivirus taking the sequence of shUSP10 or sh-NC. The infected cells were screened out by puromycin and used to construct the subcutaneous xenograft model. In the GEM-treated groups, each nude mouse was injected with GEM intraperitoneally according to the dosage of 50 mg/kg twice a week.

### CO-IP assay

The cells were lysed by IP lysis buffer (R0100, Solarbio, China). The samples were centrifuged, and supernatant was obtained. 20 μl protein A/G agarose beads (sc2003, Santacruz, USA) were put into every tube and incubated at 4 °C for 30 min. The protein concentration was measured, and the total protein was calculated. The primary antibody and 60 μl protein A/G agarose beads were put into each sample and incubated at 4 °C overnight. Then, samples were centrifuged, and supernatant was discarded. Next, the samples were washed by IP lysis buffer for three times. At last, the samples were heated at 99 °C for 8 min, and the supernatant was obtained for further utilization.

### GST pull-down assay

The plasmids GST or GST-USP10 were transformed into E.coli BL21(DE3, C1400, Solarbio, China) for amplification. The IPTG (I8070, Solarbio, China) was used to induce the expression of GST or GST-USP10 protein. Then, the E.coli BL21(DE3) was broken by ultrasound, and the supernatant was reserved after centrifugation. The Glutathione Sepharose 4B (17075601, Cytiva, USA) was put into the supernatant and incubated at 4 °C overnight. The precipitation was reserved after centrifugation and washed by NETN buffer three times. When incubated with recombinant PLK1(rPLK1, HY-P76550, MCE, USA) protein, Glutathione Sepharose 4B was resuspended by NETN buffer. And 1 μg rPLK1 was put into every group as required, and incubated at 4 °C for 4 h. Then the precipitation was washed by NETN buffer for three times. The precipitation with loading buffer was heated at 99 °C for 10 min, and the supernatant was reserved for subsequent assays. The components of NETN buffer were shown in Supplementary Table 5.

### In vivo deubiquitination assay

Flag-PLK1 was co-transfected with HA-Ub, Myc-vector, Myc-USP10, or Myc-USP10 (C424A) into HEK293 cells. The cells were treated with DMEM containing 20 μM MG132. Then the cells were harvested and treated with 200 μl SDS lysis buffer (abs9117, absin, China). The samples were boiled for 8 min, and diluted with 0.8 ml IP lysis buffer. The antibody against Flag and protein A/G agarose (sc-2003, Santacruz, USA) was added into supernatant after centrifugation. Then, the samples were rotated at 4 °C for 8 h. The precipitation was reserved and washed by IP lysis buffer for three times and was boiled with loading buffer for 10 min. At last, the supernatant was reserved after centrifugation for western blotting.

### In vitro deubiquitination assay

We first extracted the ubiquitinated Flag-PLK1 protein from HEK293 cells. In brief, Flag-PLK1 was co-transfected with HA-Ub into HEK293 cells, and the cells were cultured for 40 h. After being treated with DMEM containing 20 μM MG132, the cells were lysed by IP lysis buffer (R0100, Solarbio, China). The ubiquitinated Flag-PLK1 was purified from the cell lysate by Flag antibody and Protein A/G agarose beads. Then the beads were washed by IP lysis buffer three times. Next, we obtained solution of GST or GST-USP10 from E. coli BL21(DE3 C1400, Solarbio, China). GST or GST-USP10 plasmids were transformed into E. coli BL21(DE3). After being treated with IPTG for 8 h, the cells were broken by ultrasound. The Glutathione Sepharose 4B (17075601, Cytiva, USA) was used to purify the protein of GST or GST-USP10. Then, the GST or GST-USP10 protein was eluted by 20 mM glutathione. At last, the ubiquitinated Flag-PLK1 bound with Protein A/G agarose was incubated with eluted GST or GST-USP10 in deubiquitination buffer at 37 °C for 2 h. The precipitation was reserved and boiled with loading buffer for 10 min, and the supernatant was reserved after centrifugation for further western blotting. The components of deubiquitination buffer were shown in Supplementary Table 6.

### TUNEL assay

The reagent of TUNEL was provided by Beyotime (C1088, China). The process of the assay was performed as previous study, described [31]. At last, the IX81 fluorescence inverted microscope (Olympus, Japan) was used to collect images.

### Assay for autophagy

The PDAC cells were infected with lentivirus carrying the sequence of Mcherry-EGFP-LC3B. Then the cells were put into 24-well plate with coverslip. After cell adhesion, the cells were treated according to the requirements for 24 h. And the cells were washed and drilled, then incubated with DAPI for 15 min. At Last, the coverslips were sealed and the images were collected by confocal microscope (FV1200, Olympus, Japan).

### Transmission electron microscope

The cells were treated according to the experimental design. Then they were washed, digested, centrifuged. And the precipitation was reserved and treated with 2.5% glutaraldehyde. Next, the samples were sent to the Transmission Electron Microscopy Core at Tianjin Medical University General Hospital for further processing. Once the slices were obtained, the data were collected by transmission electron microscope (HITACHI, Japan).

### IC50

The cells were put into 96-well plate and treated with GEM after 24 h. Then the cells were treated for 48 h with different GEM concentrations, which were 0 nM, 1 nM, 10 nM, 100 nM, 1 μM, 10 μM, 100 μM. Then, the CCK8 reagent (HY-K0301, MCE, USA) was prepared according to the instructions and added to the 96-well plate. After incubation at 37 °C for 2 h, the OD value at 450 nm was measured. The GraphPad Prism 9.0 was used to calculate the IC50 values according to the values of OD 450.

### Statistical analysis

GraphPad Prism 9.0 was used to perform statistical calculations. The method of unpaired T-test and one-way ANOVA was used to compare the differences between two groups and multiple groups, respectively. For CCK8 assays, two-way ANOVA was used to perform calculation. The method of log-rank test was used to perform survival analysis. Data was expressed as the mean ± standard deviation (SD). A value of *P* < 0.05 was considered statistically significant. All experiments were performed independently at least three times.

## Results

### USP10 is upregulated in PDAC samples and associated with unfavorable prognosis

**Fig. 1:**
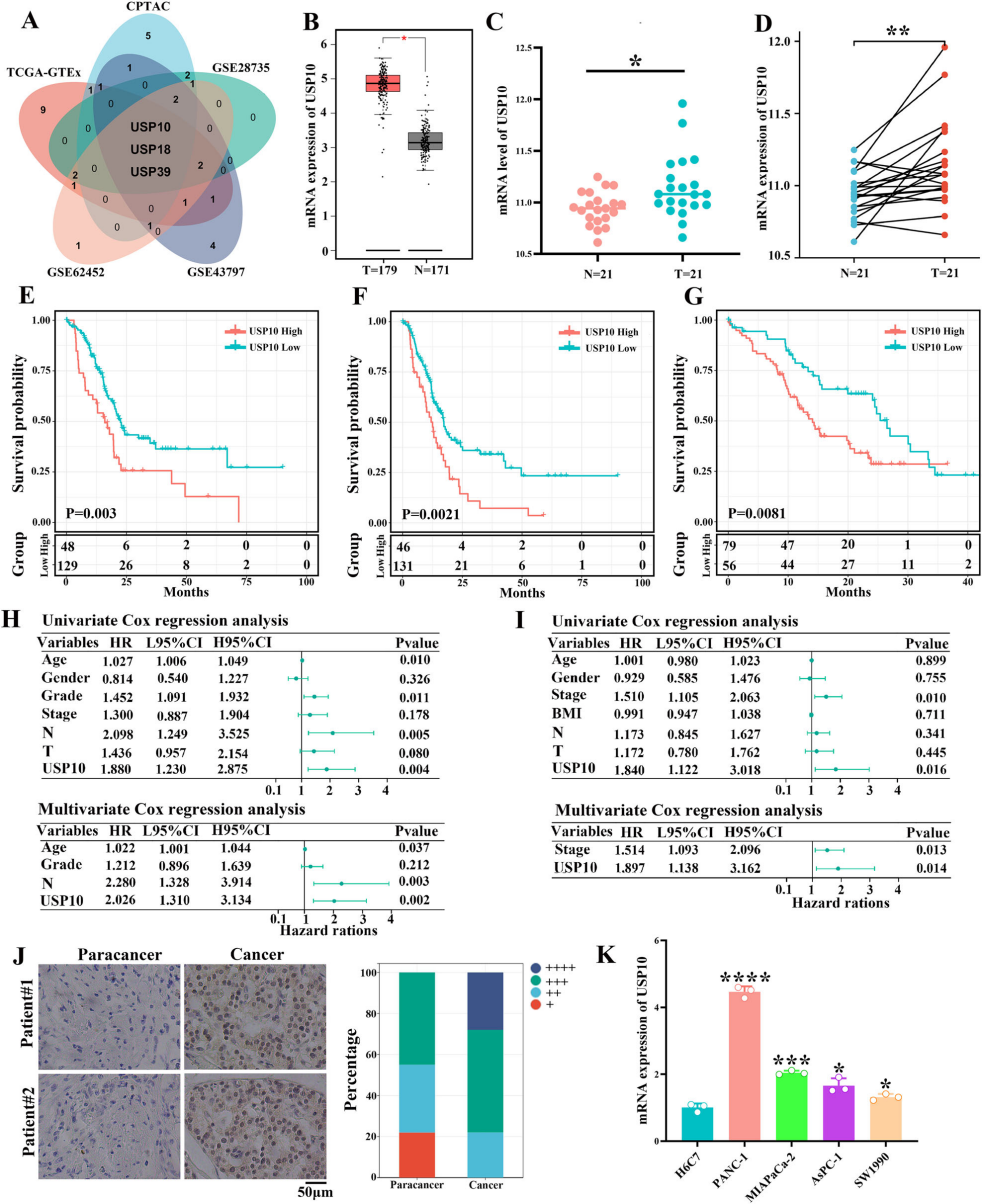
USP10 is upregulated in PDAC samples and associated with unfavorable prognosis. **A** A Venn diagram showing that USP10, USP18, and USP39 are differentially expressed in five public databases. **B** The difference of USP10 expression level between PDAC tissues and normal pancreatic tissues in TCGA-GTEx cohort. **C** The difference of USP10 expression level between PDAC tissues and paracancer tissues in CPTAC database. **D** The expression of USP10 in paired PDAC tissues and paracancer tissues. The overall survival (OS) (**E**) and progression free survival **F** in TCGA cohort showed a negative correlation with the expression of USP10. **G** The expression of USP10 was negatively correlated to the OS in CPTAC cohort. Univariate and multivariate Cox regression analysis between USP10 and OS in TCGA cohort **(H**) and CPTAC cohort (**I**)**. J** Representative images of USP10 IHC staining in PDAC tissues and paracancer tissues. **K** The expression level of USP10 in four PDAC cells was detected via RT-qPCR. *P < 0.05, **P < 0.01, ***P < 0.001, ****P < 0.0001.

We explored the mRNA expression levels of the USP family members using public PDAC databases. There were 21 differentially expressed USPs in the TCGA-GTEx database (sFig. 1A), 17 differentially expressed USPs in the CPTAC database (sFig. 1B), 14 differentially expressed USPs in the GSE62452 database (sFig. 1C), 12 differentially expressed USPs in the GSE28735 database (sFig. 1D), and 16 differentially expressed USPs in the GSE43797 database (sFig. 1E). USP10, USP18, and USP39 were differentially expressed in these five databases (Fig. 1A). Compared with normal tissues from the GTEx database, USP10 mRNA was upregulated in PDAC tissues from TCGA cohort (Fig. 1B). Compared to paracancerous tissues, USP10 mRNA was upregulated in PDAC tissues of CPTAC database (Fig. 1C, D, *P* < 0.05). In addition, USP10 mRNA was negatively associated with overall survival (OS) and progression-free survival (PFS) in the TCGA database (Fig. 1E, F). Besides, USP10 mRNA was negatively associated with overall survival (OS) in the CPTAC database (Fig. 1G). USP18 mRNA was not associated with OS and PFS in TCGA and CPTAC databases (sFig. 1F–H). Although USP39 mRNA was associated with OS and PFS in TCGA database (sFig. 1I, J), it was not associated with OS in the CPTAC database (sFig. 1K). Based on the above analysis, USP10 should be further explored in PDAC. Univariate and multivariate Cox regression analyses revealed that USP10 was an independent prognostic factor for OS (Fig. 1H) and PFS (sFig. 1L, M) in the TCGA database and an independent prognostic factor for OS in the CPTAC databases (Fig. 1I). Next, IHC was used to detect the protein expression level of USP10 in cancerous and para-cancerous tissues from 18 PDAC patients of our cohort. The results suggested that the USP10 protein was upregulated in PDAC compared to that in tissues adjacent to cancer (Fig. 1J). Compared to normal pancreatic ductal epithelial cells, H6C7, USP10 mRNA was upregulated in the four PDAC cell lines (Fig. 1K). In summary, our data suggested that USP10 was upregulated in PDAC and contributed to an unfavorable prognosis.

### USP10 promotes the malignant biological behaviors of PDAC cells

**Fig. 2:**
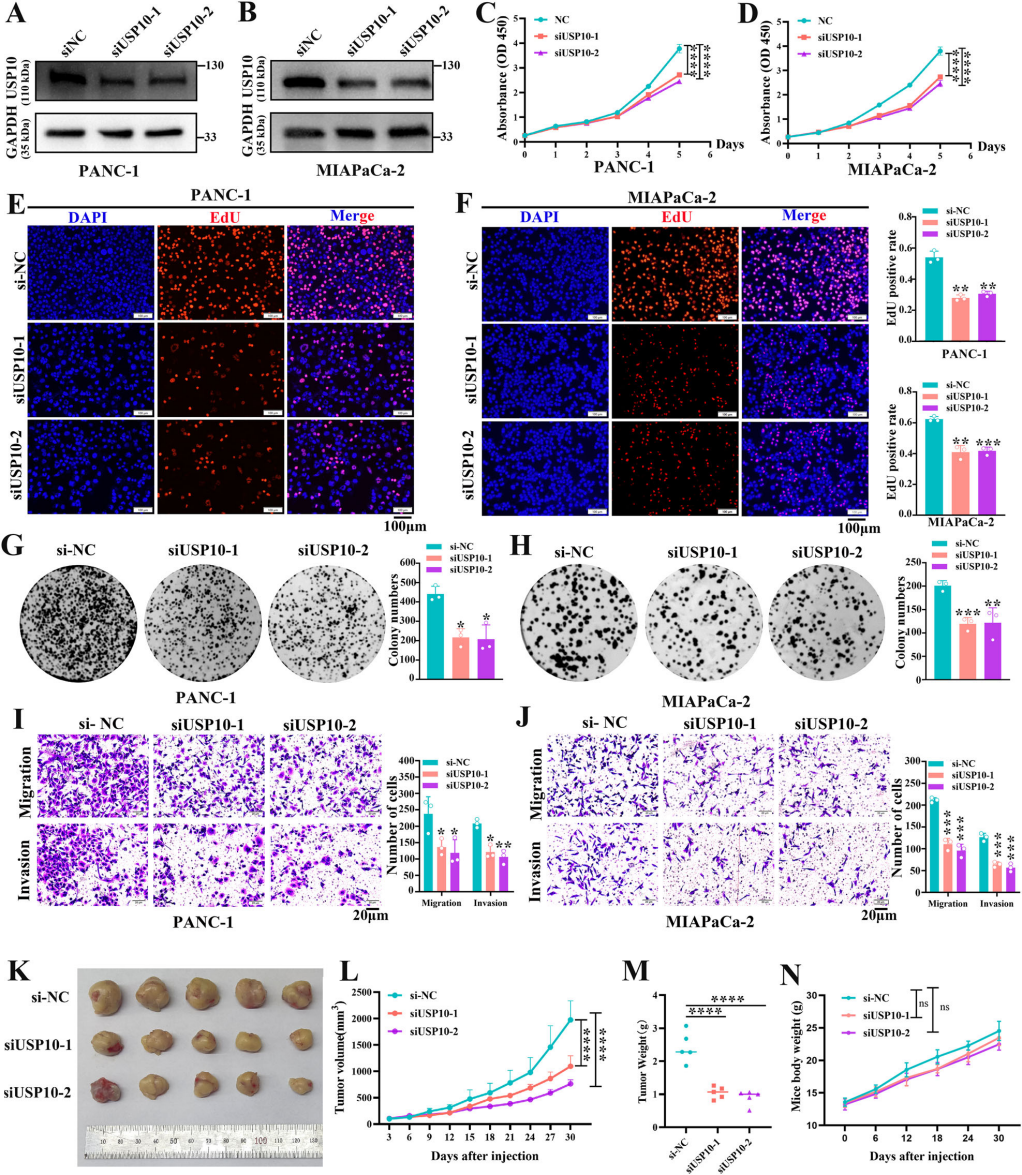
Biological functions of USP10 in PANC-1 and MIAPaCa-2 cells. **A**, **B** The verification of USP10 siRNAs knockdown efficiency. **C**, **D** The effect of USP10 knockdown on proliferation detected through CCK-8 assays. **E**, **F** The change of proliferation after USP10 knockdown detected via EdU assays. **G**, **H** The influence of USP10 knockdown on the colony formation ability. **I**, **J** Transwell assays revealed decreased migration and invasion after USP10 knockdown. **K** Image of tumors in xenograft models following siRNA treatment. **L**, **M** Curves of tumor growth and weight for animal study. **N** Mice body weight among the groups. Data are presented as mean ± sd. from three biologically independent samples. **P < 0.01, ***P < 0.001, ****P < 0.0001.

We investigated the role of USP10 in PDAC cells. Four PDAC cell lines exhibited varying expression levels of USP10. PANC-1 and MIAPaCa-2 cells showed the highest expression, whereas SW1990 cells showed the lowest (Fig. 1K). Therefore, we knocked down USP10 in PANC-1 and MIAPaCa-2 cells and overexpressed USP10 in SW1990 cells. To further study the function of the USP10 gene, we overexpressed USP10 in SW1990 cells using the plasmid. USP10 was knocked down with small interfering RNA (siRNA) in PANC-1 and MIAPaCa-2 cells. Western blotting and qPCR were used to verify USP10 knockdown (Fig. 2A, B, and sFig. 2A–D) or over-expression (sFig. 2E, F, and 3A). The CCK-8 assay revealed that proliferation was suppressed by USP10 knockdown in PANC-1 and MIAPaCa-2 cells (Fig. 2C, D) and was promoted by USP10 overexpression in SW1990 cells (sFig. 3B). The EdU assay revealed that the proliferative rate of PDAC cells was reduced by USP10 knockdown in PANC-1 and MIAPaCa-2 cells (Fig. 2E, F) and was increased by USP10 overexpression in SW1990 cells (sFig. 3C). The knockdown of USP10 in PANC-1 and MIAPaCa-2 cells resulted in a decrease in colony formation (Fig. 2G, H), whereas the overexpression of USP10 in SW1990 cells led to an increase in colony formation ability (sFig. 3D). As observed in the Transwell assay, the migration and invasion capacities of PANC-1 and MIAPaCa-2 cells were inhibited by USP10 knockdown (Fig. 2I, J). Conversely, the overexpression of USP10 in SW1990 cells enhanced their migration and invasion abilities (sFig. 3E). The results of the wound healing assay indicated that the migration rate was reduced upon USP10 knockdown in PANC-1 and MIAPaCa-2 cells (sFig. 3F, G), whereas it was enhanced after USP10 overexpression in SW1990 cells (sFig. 3H). We explored the biological functions of USP10 in vivo. The findings demonstrated that the control group exhibited a higher growth rate and tumor weight than the USP10 knockdown group (Fig. 2K–M). Besides, there was no difference in mice body weight among the groups (Fig. 2N). In conclusion, USP10 promoted PDAC progression both in vivo and in vitro.

### USP10 interacts with PLK1

**Fig. 3:**
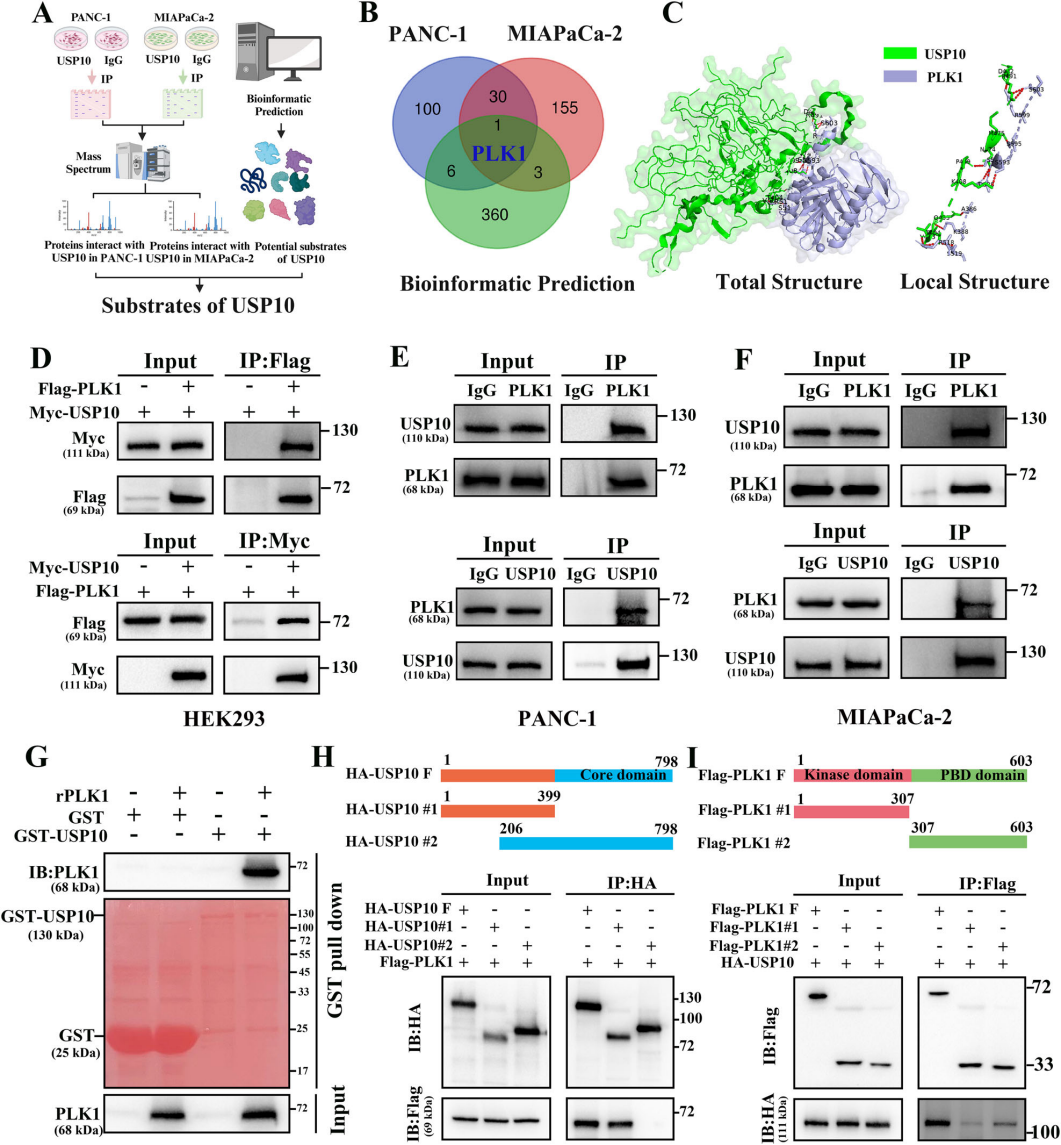
USP10 directly interacts with PLK1. **A** Flow chart of the USP10 substrate screening process. **B** A Venn diagram showing that PLK1 is the only substrate of USP10. **C** The interaction between USP10 and PLK1 was confirmed through molecular docking analysis. **D** Exogenous CO-IP assay of USP10 and PLK1 in HEK293 cells. **E**, **F** Endogenous interaction of USP10 and PLK1 detected via Co-IP assay in PDAC cells. **G** The direct interaction between USP10 and PLK1 was validated using a GST pull-down assay. **H** HEK293 cells were transfected with the respective plasmids, and full length and fragments of USP10 were used to pull down full-length PLK1. **I** HEK293 cells were transfected with the respective plasmids, and full length and fragments of PLK1 were used to pull down full-length USP10.

To further investigate the functional mechanism of USP10, we identified its substrates as shown in Fig. 3A. In immunoprecipitation-mass spectrometry (IP-MS) analysis, USP10 in PANC-1 and MIAPaCa-2 cells was purified using the IP method, and the shotgun technique was employed to analyze the proteins interacting with USP10 protein (sFig. 4A, B). IP-MS assay results revealed distinct interactions between USP10 and 137 proteins in PANC-1 cells, whereas 189 proteins exhibited specific interactions with USP10 in MIAPaCa-2 cells. On the other hand, bioinformatics prediction (http://ubibrowser.bio-it.cn/ubibrowser_v3/) showed that 370 proteins may be substrates for USP10. PLK1 was identified as a potential substrate of USP10 by integrating the above findings (Fig. 3B). The IP-MS results showed that two PLK1 unique peptides were detected in both PANC-1 and MIAPaCa-2 cells (sFig. 4C, D). Furthermore, molecular docking revealed that USP10 interacted with PLK1 protein (Fig. 3C). CO-IP assays were performed to confirm the interaction between USP10 and PLK1. Our findings demonstrated that exogenous USP10 interacted with exogenous PLK1 in HEK293 cells (Fig. 3D). In addition, the endogenous interaction between USP10 and PLK1 was confirmed using CO-IP assays in PANC-1 and MIAPaCa-2 cells (Fig. 3E, F). The results of the GST pull-down assay demonstrated that USP10 directly interacted with PLK1 protein (Fig. 3G). To further explore the domains of USP10 that interact with PLK1, we constructed full-length and mutant plasmids of USP10 and PLK1 and transferred them into HEK293 cells. The results showed that 1-206 amino acids of USP10 can interact with PLK1 (Fig. 3H). Similarly, 307-603 amino acids of PLK1 can interact with USP10 (Fig. 3I). In summary, our findings suggested that USP10 directly interacted with PLK1.

### USP10 maintains PLK1 stability by triggering deubiquitination of PLK1

**Fig. 4:**
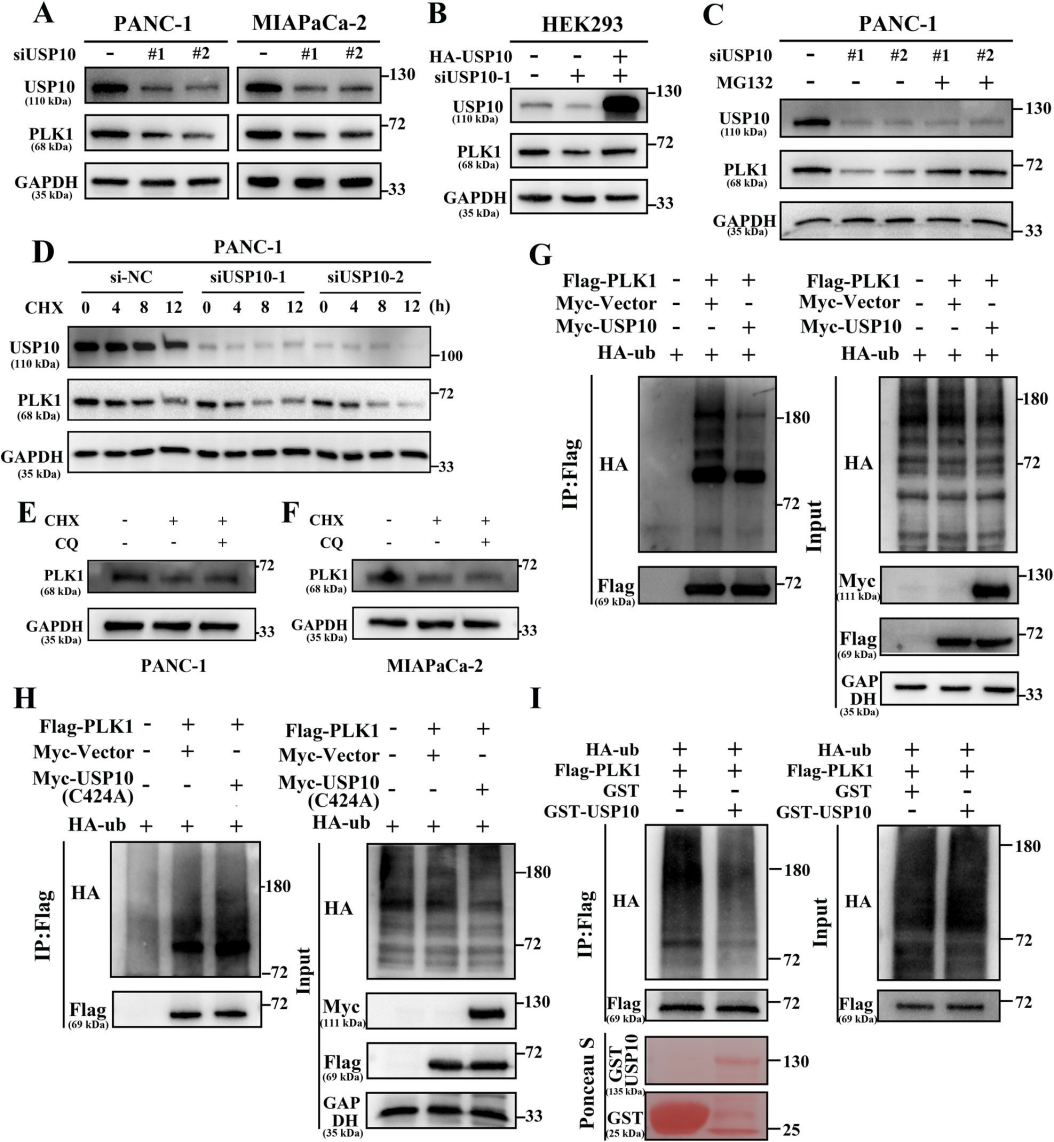
USP10 maintains PLK1 protein stability by triggering deubiquitination of PLK1. **A** The change of USP10 and PLK1 protein after transfection with USP10 siRNAs in PDAC cells. **B** The protein level of USP10 and PLK1 were detected via western blot after transfection with HA-USP10 plasmids. **C** PANC-1 cells were transfected with si-NC, siUSP10-1, and siUSP10-2 and treated with 20 μM MG132 for 24 h, and the protein level of USP10 and PLK1 were detected through western blot. **D** PANC-1 cells were transfected with different siRNAs and treated with 10 μg/ml CHX for 0 h, 4 h, 8 h, and 12 h. The change of USP10 and PLK1 proteins were detected. The PANC1 **E** and MIAPaCa-2 **F** cells were treated with protein synthesis inhibitor CHX and autophagy inhibitor CQ, and the protein level of PLK1 was detected. **G** Myc-USP10 and other plasmids were transfected into HEK293 cells and treated with 20 μM MG132 for 6 h. The ubiquitination level of PLK1 was detected via IP assay. **H** Myc-USP10 (C424A) and other plasmids were transfected into HEK293 cells and treated with 20 μM MG132 for 6 h. The ubiquitination level of PLK1 was detected via IP assay. **I** The ubiquitinated Flag-PLK1 was purified from HEK293, and GST-USP10 was purified from E. coli BL21 (DE3). Next, the two proteins were incubated in the deubiquitination buffer at 37 °C for 2 h. The ubiquitination level of Flag-PLK1 was detected through western blot.

USP10 increases the stability of substrate proteins depending on its function as a deubiquitinating enzyme. Therefore, we hypothesized that USP10 increased the stability of PLK1. To verify our hypothesis, we knocked down USP10 in PDAC cells and observed a corresponding decrease in PLK1 protein (Fig. 4A and sFig. 5A). Moreover, this effect was rescued by the overexpression of exogenous USP10 in HEK293 cells (Fig. 4B and sFig. 5B). In contrast, the effect of USP10 on the stability of PLK1 disappeared after treatment with the proteasome inhibitor MG132 in PANC-1 cells (Fig. 4C and sFig. 5C). After treatment with the protein synthesis inhibitor cycloheximide (CHX), USP10 knockdown significantly increased PLK1 degradation (Fig. 4D and sFig. 5D). After treated with CHX in PANC-1(Fig. 4E) and MIAPaCa-2 (Fig. 4F) cells, the PLK1 protein level did not change whether autophagy inhibitor (chloroquine, CQ) was used or not. These results suggested that the stability of PLK1 protein regulated by USP10 might depend on the ubiquitin-proteasome pathway.

To investigate the effect of USP10 on the ubiquitination level of PLK1, we performed deubiquitination assays in vivo and in vitro. The results showed that USP10 protein (Fig. 4G), but not its inactive mutant USP10 C424A (Fig. 4H), could deubiquitinate the PLK1 protein. The results of the deubiquitination assay in vitro showed that USP10 directly deubiquitinated the PLK1 protein (Fig. 4I). Thus, USP10 could maintain the stability of PLK1 by triggering deubiquitination of PLK1.

### PLK1 promotes the malignant biological behaviors of PDAC cells

**Fig. 5:**
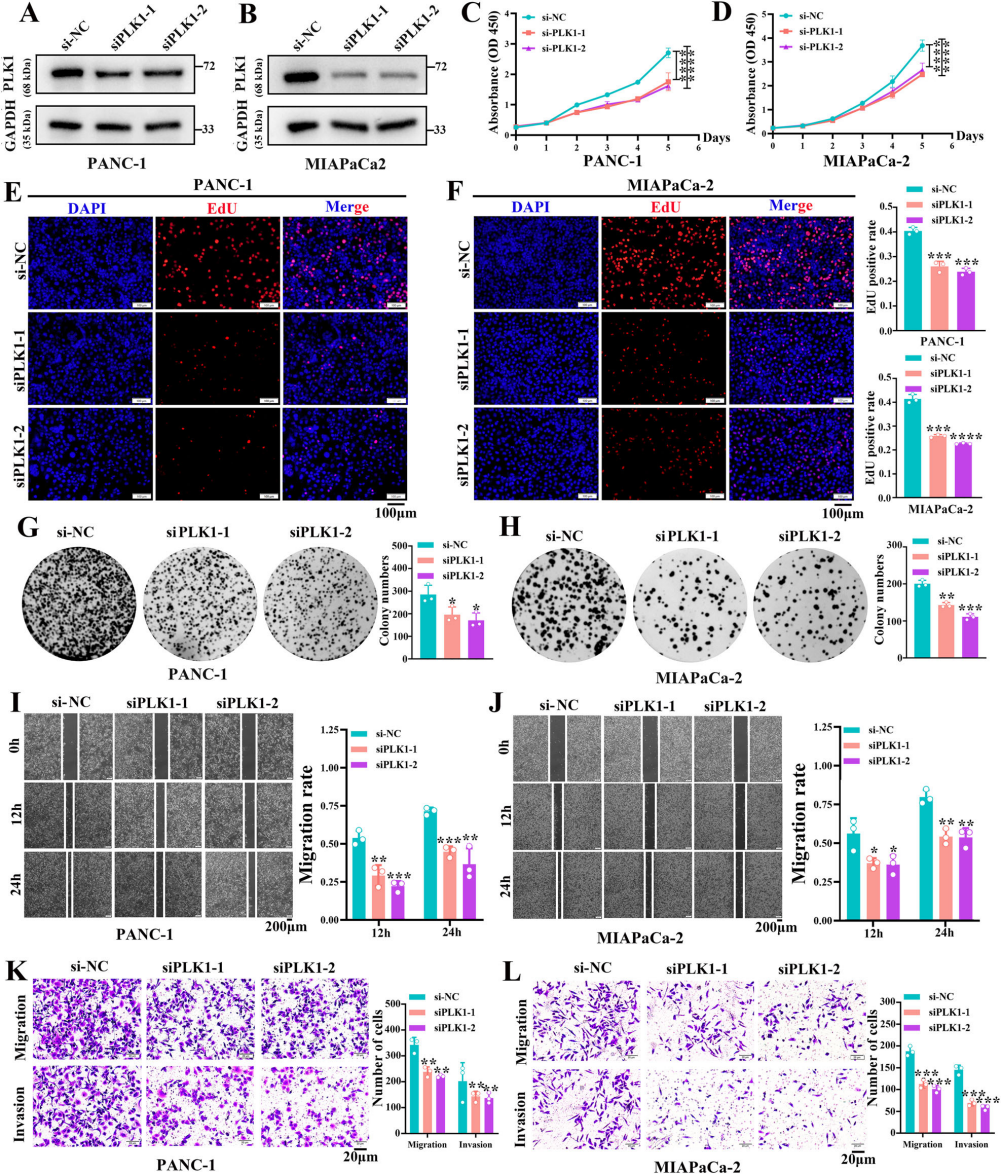
Biological functions of PLK1 in PANC-1 and MIAPaCa-2 cells. **A**, **B** Confirmation of PLK1 knockdown efficiency. **C**, **D** CCK-8 assay was used to assess the impact of PLK1 knockdown on the proliferation. **E**, **F** EdU assay was employed to assess the impact of PLK1 knockdown on the proliferation. **G**, **H** Influence of PLK1 knockdown on the colony formation ability. **I**, **J** The change in migration ability after PLK1 knockdown confirmed through wound healing assay. **K**, **L** Influence of PLK1 knockdown on the migration and invasion ability, detected using Transwell assay. Data are presented as mean ± sd. from three biologically independent samples. *P < 0.05, **P < 0.01, ***P < 0.001, ****P < 0.0001.

We demonstrated that PLK1 was a substrate of USP10 and subsequently investigated the role of PLK1 in the biological behaviors of PDAC cells. We constructed siRNAs and overexpression plasmid of PLK1. The results indicated that the mRNA and protein expression of PLK1 were decreased by siRNAs of PLK1 in PANC-1 (Fig. 5A and sFig. 6A, B) and MIAPaCa-2 cells (Fig. 5B and sFig. 6C, D). Additionally, the mRNA and protein expression of PLK1 were increased by PLK1 overexpression in SW1990 cells (sFig. 6E, F and 7A).

The CCK-8 assays revealed that the proliferation of PANC-1 (Fig. 5C) and MIAPaCa-2 (Fig. 5D) cells was suppressed upon PLK1 knockdown, whereas SW1990 cell proliferation was enhanced upon PLK1 overexpression (sFig. 7B). The EdU assays revealed that the proliferation rate of PANC-1 (Fig. 5E) and MIAPaCa-2 (Fig. 5F) cells was reduced by the knockdown of PLK1, whereas the proliferation rate of SW1990 cells was increased by the overexpression of PLK1 (sFig. 7C, D). The inhibition of PLK1 decreased the colony formation ability of PANC-1 (Fig. 5G) and MIAPaCa-2 (Fig. 5H) cells, whereas the overexpression of PLK1 increased the colony formation ability of SW1990 cells (sFig. 7E, F). The migration ability was inhibited by the knockdown of USP10 in PANC-1 (Fig. 5I) and MIAPaCa-2 (Fig. 5J) cells, whereas the migration ability of SW1990 cells was promoted by the overexpression of USP10 (sFig. 7G). As revealed by Transwell assays, the invasion ability could be decreased by the knockdown of USP10 in PANC-1 (Fig. 5K) and MIAPaCa-2 (Fig. 5L) cells. However, the invasive ability was enhanced by USP10 upregulation in SW1990 cells (sFig. 7H). In conclusion, PLK1 promotes the malignant behavior of PDAC cells.

### The effect of USP10 overexpression on malignant behavior of PDAC cells can be partially rescued by PLK1 knockdown

We next explored whether the effect of USP10 on the malignant biological behavior of PDAC cells was dependent on PLK1. USP10 was successfully knocked down and PLK1 was successfully overexpressed in PANC-1 (sFig. 8A) and MIAPaCa-2 (sFig. 8B) cells. The CCK-8 and EdU assays showed that the increase of proliferation induced by USP10 overexpression can be partially rescued by PLK1 knockdown in PANC-1(sFig. 8C, E) and MIAPaCa-2 cells (sFig. 8D, F). The Transwell assays showed that the increase of migration and invasion induced by USP10 overexpression can be partially rescued by PLK1 knockdown in PANC-1(sFig. 8G) and MIAPaCa-2 cells (sFig. 8H). The above results showed that the effect of USP10 on the malignant biological behavior of PDAC cells was partially dependent on PLK1.

### USP10 affects the autophagy of PDAC cells partially through PLK1

**Fig. 6:**
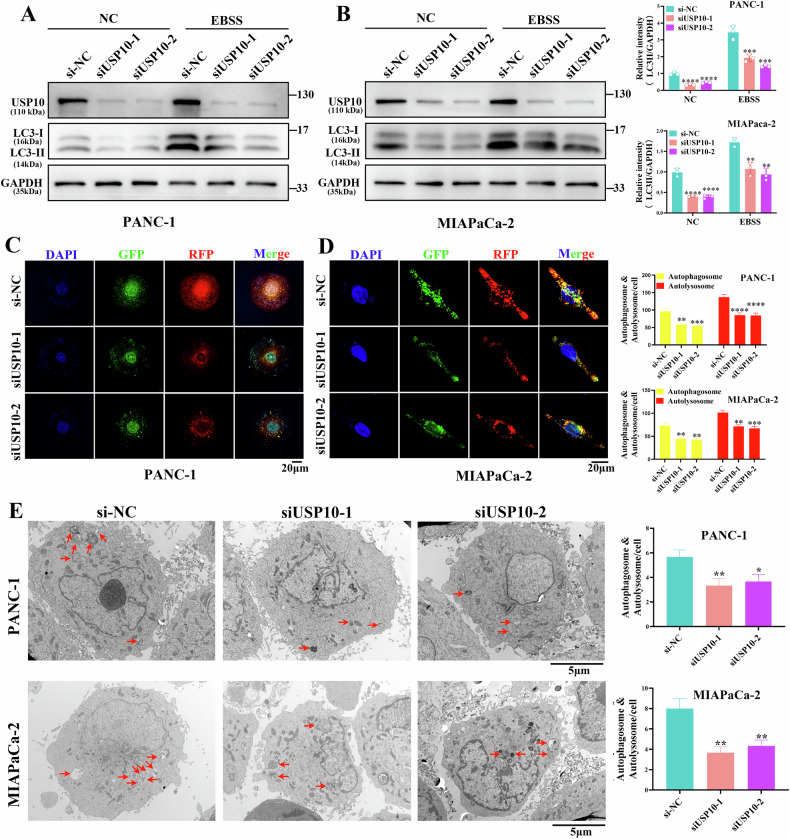
USP10 affects the autophagy of PDAC cells. **A**, **B** The si-NC, siUSP10-1, and siUSP10-2 were transfected into PDAC cells. When autophagy needed to be activated, the cells were treated with EBSS medium for 8 h. Subsequently, the autophagy-related proteins were detected through western blot. **C**, **D** PDAC cells were infected with the lentivirus Mcherry-EGFP-LC3B. Next, USP10 was knocked down, and the cells were treated with EBSS medium for 8 h before being observed under a confocal microscope. **E** The PDAC cells were treated with EBSS medium for 8 h following USP10 knockdown and were observed using transmission electron microscope. Data are presented as mean ± sd. from 3 biologically independent samples. *P < 0.05, **P < 0.01, ***P < 0.001, ****P < 0.0001.

To further explore the biological processes involving USP10, USP10-related genes were identified from TCGA PDAC data using the criteria of |R| > 0.3, *P* < 0.05. A total of 3137 genes were identified as USP10-related genes, and the detailed information was shown in Supplementary Table 7. Kyoto Encyclopedia of Genes and Genomes enrichment analysis indicated that USP10 may participate in the regulation of the animal autophagy pathway (sFig. 9A). In the biological process enrichment analysis of Gene Ontology, the regulation of macroautophagy was enriched (sFig. 9B). Our findings suggested that USP10 participated in the regulation of autophagy. Therefore, we investigated the role of USP10 in autophagy in PDAC. The detection of autophagy-related proteins showed that the ratio of LC3-II/GAPDH decreased after USP10 knockdown both at the basal and active stages of autophagy (Fig. 6A, B). In addition, the Beclin1 protein was decreased and the p62 protein was increased after USP10 knockdown, both at the basal and active stages of autophagy (sFig. 10A, B). The above results showed that USP10 could promote autophagy. Next, we infected PDAC cells with lentivirus Mcherry-EGFP-LC3B and used Earle’s balanced salt solution (EBSS) to activate autophagy. The results showed that autolysosomes marked with red puncta and autophagosomes marked with yellow decreased after USP10 knockdown in PDAC cells (Fig. 6C, D). Transmission electron microscopy analysis suggested that autophagic vesicles decreased after USP10 knockdown in PDAC cells (Fig. 6E). USP10 knockdown inhibited autophagy in PDAC cells and reduced PLK1 protein stability (Fig. 4A, D). The previous studies showed that PLK1 could positively regulates autophagy [18, 33, 34]. Therefore, we hypothesized that USP10 regulates autophagy through PLK1.

**Fig. 7:**
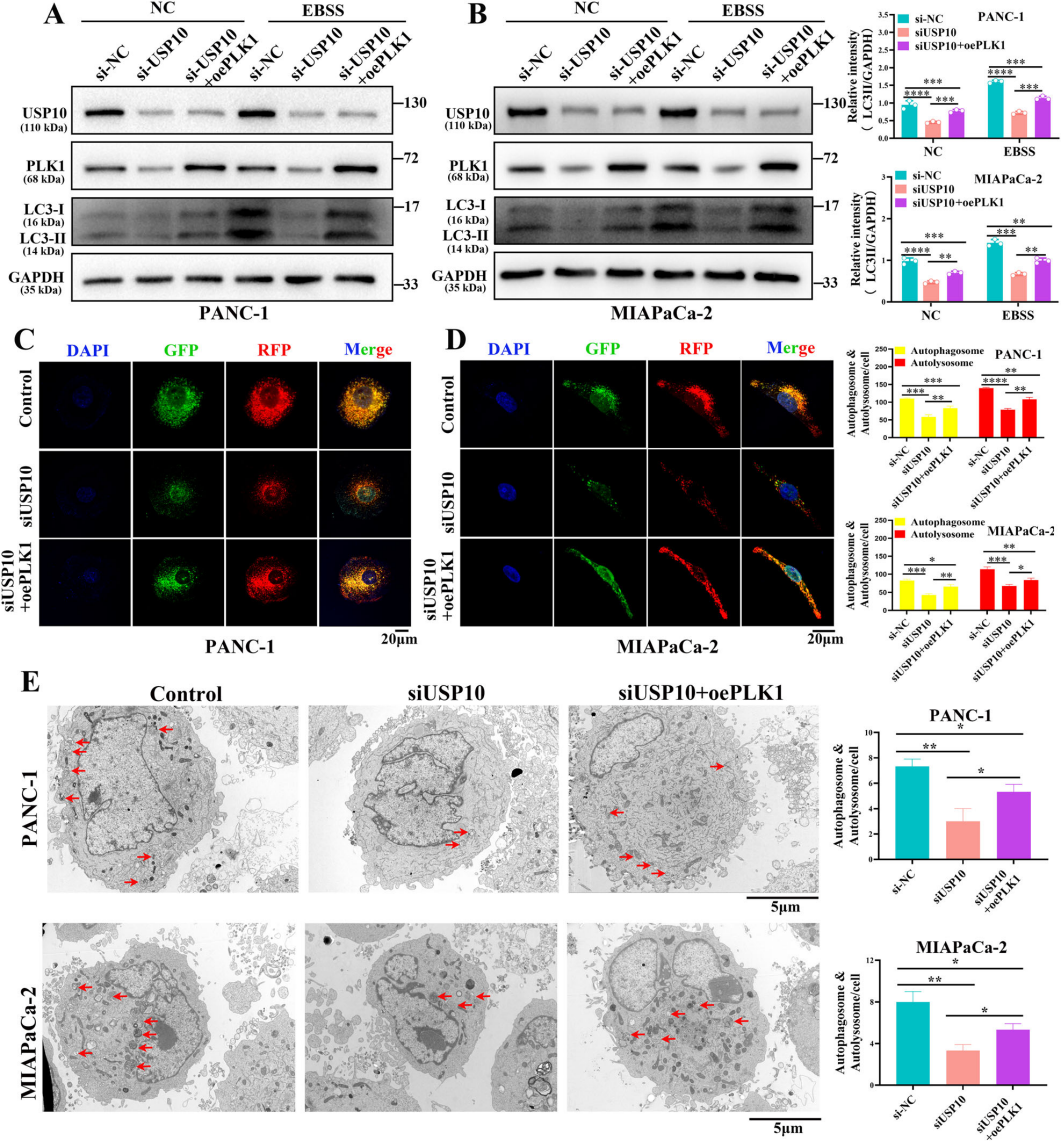
USP10 affects the autophagy of PDAC cells partially through PLK1. **A**, **B** The si-NC, siUSP10-1, siUSP10-2, and the overexpression plasmid of PLK1 were transfected into PDAC cells as required. The EBSS medium was used to activate autophagy. The autophagy-related proteins were detected. **C**, **D** PDAC cells were infected with the lentivirus Mcherry-EGFP-LC3B. Next, si-USP10 and the overexpression plasmid of PLK1 were transfected into PDAC cells as required, and the cells were treated with EBSS medium for 8 h before being observed under a confocal microscope. **E** si-USP10 and the overexpression plasmid of PLK1 were transfected into PDAC cells according to the requirement, and the cells were treated with EBSS medium for 8 h before being observed under a transmission electron microscope. *P < 0.05, **P < 0.01, ***P < 0.001, ****P < 0.0001.

We performed rescue assays to test this hypothesis. Our findings suggested that the decrease in the LC3-II/GAPDH ratio induced by USP10 downregulation was partially rescued by PLK1 overexpression in PDAC cells (Fig. 7A, B). In addition, the decrease of Beclin1 protein and the increase of p62 protein induced by USP10 downregulation were partially rescued by PLK1 overexpression in PDAC cells (sFig. 10C, D). Furthermore, the decrease in the red and yellow puncta after USP10 knockdown was partially rescued by PLK1 overexpression in PDAC cells (Fig. 7C, D). Transmission electron microscopy revealed that the decrease in autophagic vesicles induced by USP10 knockdown was partially increased by PLK1 overexpression in PDAC cells (Fig. 7E). These results suggest that USP10 partially promotes autophagy in PDAC cells through PLK1.

### USP10 affects the chemotherapy sensitivity of PDAC cells to GEM partially through autophagy

**Fig. 8:**
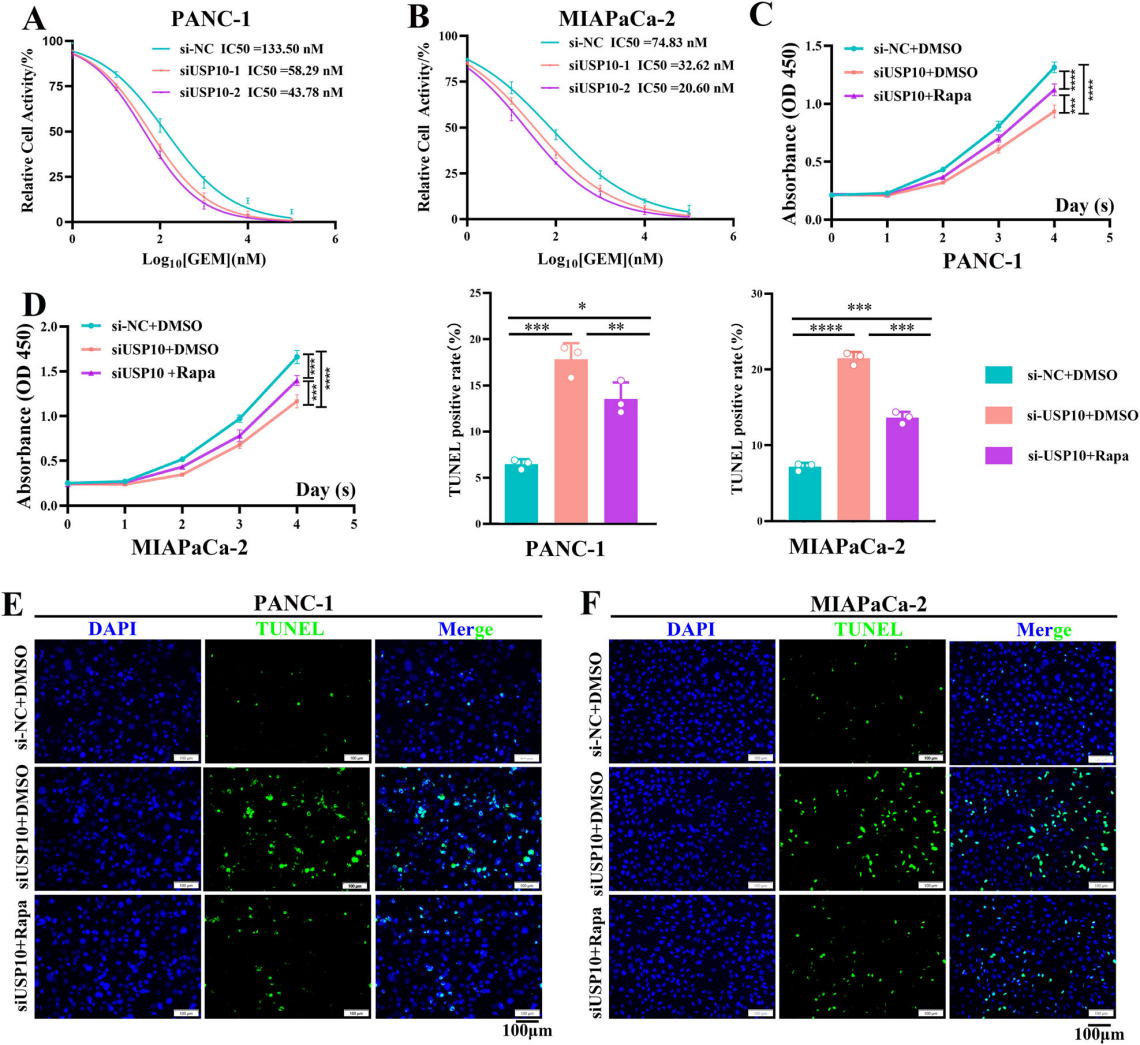
USP10 affects the chemotherapy sensitivity of PDAC cells to gemcitabine (GEM) partially through autophagy. **A**, **B** After USP10 knockdown, the IC50 of GEM for PANC-1 and MIAPaCa-2 cells was determined by treating the cells with different concentrations of GEM. **C**, **D** The PANC-1 and MIAPaCa-2 cells were treated with GEM after USP10 knockdown. The proliferation was detected through CCK-8 assay after the cells were treated with 100 nM Rapa for 48 h, as required. **E**, **F** The PANC-1 and MIAPaCa-2 cells were treated with GEM after USP10 knockdown. TUNEL assay was used to detect the cell apoptosis after the cells were treated with 100 nM Rapa for 48 h, as required. *P < 0.05, **P < 0.01, ***P < 0.001, ****P < 0.0001.

USP10 was found to regulate autophagy in PDAC cells (Fig. 6). It has been proven that autophagy can affect the chemotherapy sensitivity of PDAC to GEM [25, 35]. Therefore, we explored the effects of USP10 on GEM chemotherapy sensitivity in PDAC. Compared to that of the si-NC group (IC50 = 133.50 nM), the IC50 value of GEM decreased after treatment with siUSP10-1 (58.29 nM) and siUSP10-2 (43.78 nM) in PANC-1 cells (Fig. 8A). Compared to the si-NC group (IC50 = 74.83 nM), the IC50 value of GEM decreased after treatment with siUSP10-1 (32.62 nM) and siUSP10-2 (20.60 nM) in MIAPaCa-2 cells (Fig. 8B). The results of IC50 showed that USP10 could reduce the sensitivity of PDAC to GEM. Next, we explored whether the effect of USP10 on GEM chemotherapy sensitivity is dependent on autophagy. PDAC cells were treated with GEM before the CCK-8 and TUNEL assays. CCK-8 assay revealed that proliferation was inhibited after USP10 knockdown in PDAC cells, and this effect was partially rescued by treatment with rapamycin (RAPA)—an autophagy activator (Fig. 8C, D). The results of the TUNEL assay showed that USP10 knockdown promoted apoptosis in PANC-1 and MIAPaCa-2 cells, and this effect was partially reversed by RAPA (Fig. 8E, F). Our findings revealed that USP10 partially attenuated the chemotherapeutic sensitivity of PDAC cells to GEM through autophagy.

### USP10 inhibition combined with GEM can synergistically inhibit PDAC progression in vivo and in vitro

**Fig. 9:**
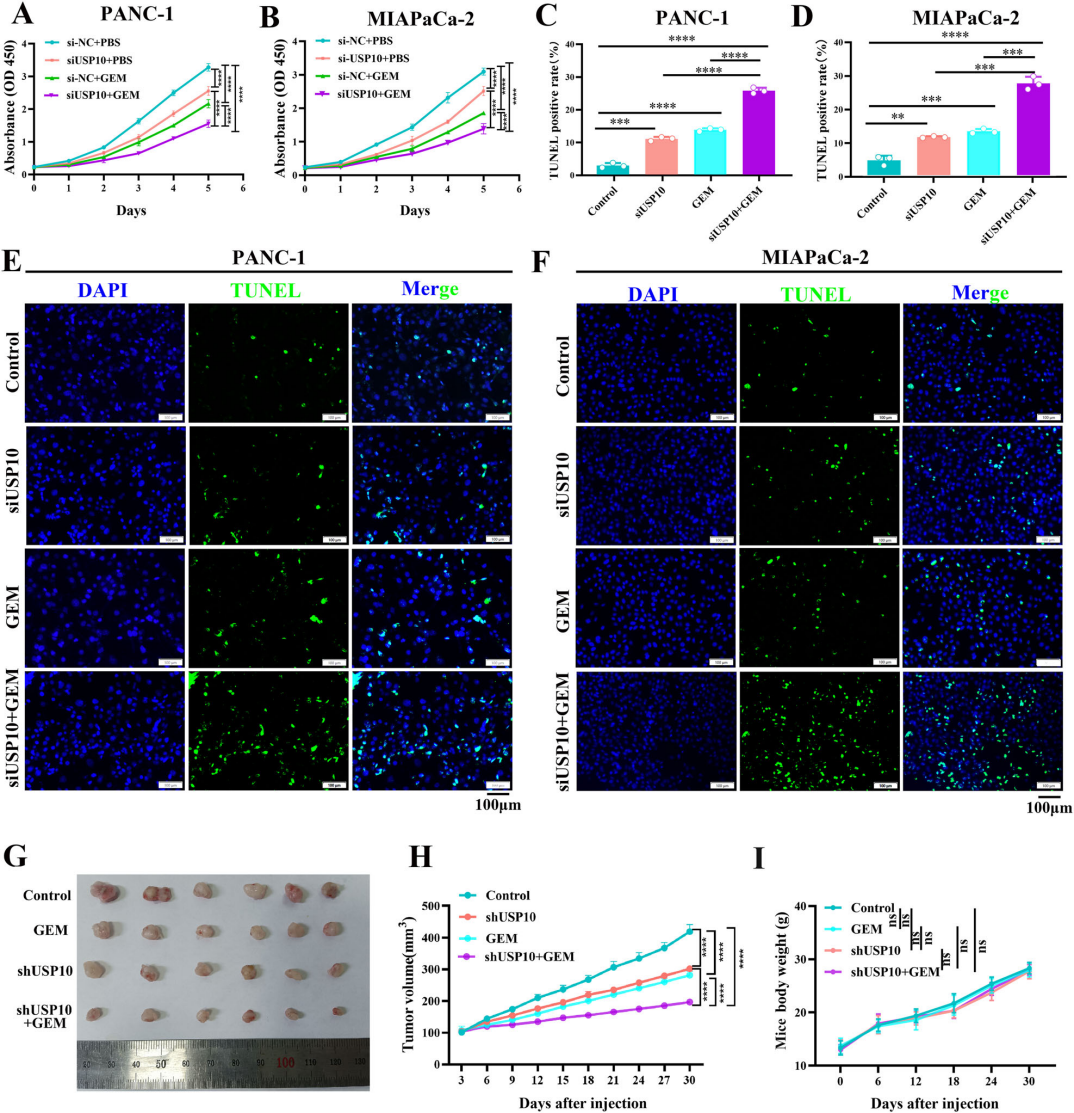
Inhibition of USP10 combined with gemcitabine (GEM) can synergistically inhibit the progress of PDAC. **A**, **B** The synergistic inhibitory effect of USP10 knockdown combined with GEM on the cell proliferation detected through CCK-8 assay. **C**–**F** The TUNEL assay demonstrated that USP10 knockdown combined with GEM had a synergistic stimulative effect, significantly enhancing cell apoptosis. **G**, **H** PANC-1 cells were used to construct xenograft tumor models. USP10 knockdown or GEM inhibited the growth of the tumors in vivo, whereas USP10 knockdown combined with GEM had synergistic inhibitory effect on tumor growth. **I** Mice body weight among the groups. Data are presented as mean ± sd. from three biologically independent samples. **P < 0.01, ***P < 0.001, ****P < 0.0001.

USP10 enhanced the chemotherapeutic resistance of GEM by partially promoting autophagy. Therefore, we hypothesized that USP10 knockdown combined with GEM treatment would synergistically inhibit PDAC progression. The CCK-8 assay revealed that the proliferation of PDAC cells was reduced by either USP10 knockdown or the application of GEM compared to that in the control group. When USP10 knockdown was combined with GEM treatment, PDAC cell proliferation was further inhibited (Fig. 9A, B). The TUNEL assay indicated that the knockdown of USP10 or application of GEM increased the apoptosis rate of PDAC cells compared to that of the control group. The apoptotic rate of PDAC cells was further increased when USP10 knockdown was combined with GEM (Fig. 9C–F). To further support our hypothesis, we explored the effects of USP10 knockdown combined with GEM in vivo. The results showed that either USP10 knockdown alone or the application of GEM could suppress the growth of the PDAC tumors. When USP10 knockdown was combined with GEM treatment, tumor growth was further suppressed (Fig. 9G, H). Besides, there was no difference in mice body weight among the groups (Fig. 9I). In summary, the inhibition of USP10 combined with GEM synergistically inhibited the progression of PDAC in vivo and in vitro.

## Discussion

PDAC is the prevailing histological subtype of PC, with more than 300,000 deaths occurring annually [36]. Deubiquitination regulates the stability of proteins that affect the progression of various cancers [37]. Exploring deubiquitination to identify potential therapeutic strategies for PDAC is an attractive approach. In this study, we found that USP10, a DUB, was upregulated in PDAC and contributed to its poor progression. In addition, we demonstrated that USP10 promoted PDAC progression in vitro and in vivo. Moreover, USP10 directly interacted with PLK1 and increased its protein stability, thereby affecting autophagy and GEM sensitivity of PDAC cells. Finally, we showed that the inhibition of USP10 combined with GEM synergistically inhibited the progression of PDAC in vivo and in vitro.

USP10 is a deubiquitinating enzyme belonging to the USP family. It is reported that USP10 could affect cancer progression in different ways. Li et al. have demonstrated that USP10 upregulates CLK2 by deubiquitinating PABPC1 to promote PDAC progression [16]. Other studies have revealed that USP10 promotes the progression of hepatocellular carcinoma, esophageal squamous cell carcinoma, and glioblastoma [38–40]. Conversely, USP10 could act as a tumor suppressor by deubiquitinating different substrates in renal cell carcinoma and lung cancer [41, 42]. The most important finding of this study was the interaction between USP10 and PLK1(Fig. 3). Our study demonstrated that USP10 acted as a tumor promoter and deubiquitinated PLK1 in PDAC (Fig. 4). A previous study suggested that PLK1 could be deubiquitinated by USP7 [19]. The current study identified USP10 as a novel deubiquitinating enzyme for PLK1, thereby expanding the deubiquitination regulatory network of PLK1.

PLK1 is a conserved Ser/Thr kinase that regulates the cell cycle [17]. Currently, numerous studies focus on the effects of PLK1 on autophagy. PLK1 co-localizes with MTORC1 in lysosomes and directly phosphorylates RAPTOR, one of the components of MTORC1. Through this process, PLK1 reduces the distribution of MTORC1 in lysosomes and promotes autophagy [18]. Another study showed that PLK1 promoted osteosarcoma cell proliferation by promoting autophagy [43]. Wang et al. revealed that suppression of PLK1 could enhance the sensitivity of breast cancer cells to radiation by inhibiting autophagy [34]. Currently, the role of USP10 in autophagy remains largely unknown in PDAC. Our study suggested that USP10 could regulate autophagy in PDAC cells through PLK1 (Figs. 6–7), providing new insights into the function of USP10 and further refining the regulatory network of autophagy.

GEM is a first-line chemotherapy drug for advanced PDAC [34], however, its effect is not ideal owing to the development of chemotherapy resistance [44]. The emergence of pro-survival autophagy is an important mechanism underlying GEM chemotherapy resistance in PDAC [25]. Wang et al. demonstrated that girdin could promote autophagy by interacting with p62 and increasing the chemotherapeutic resistance of GEM [45]. In another study, USP9x inhibition increased sensitivity to GEM chemotherapy by attenuating autophagy in PDAC cells [46]. In addition, autophagy was increased in PC cells after treatment with GEM [24]. Moreover, the antitumor effects of GEM are enhanced when autophagy is inhibited [35]. In conclusion, there is a positive feedback loop between autophagy and GEM resistance. Therefore, suppression of autophagy to increase sensitivity to GEM chemotherapy is a feasible strategy. Our study revealed that the suppression of USP10 could decrease autophagy in PDAC cells (Fig. 6) and increase the chemotherapy sensitivity of GEM in vivo and in vitro (Figs. 8, 9), providing a potential pathway to enhance the anti-tumor activity of GEM.

However, there are some limitations in our study. Firstly, this study only explored the different expression of USP10 protein between PC tissue and adjacent tissues, and did not explore the influence of USP10 protein level on the survival of PC patients. In further study, we will collect enough PC specimens to explore the effects of USP10 protein level on the prognosis of PC patients. Secondly, this study proved that USP10 could affect the ubiquitination level of PLK1, but the specific type of ubiquitination affected by USP10 has not been explored. In the further study, we will design the plasmids of ubiquitin containing different mutation sites to explore the type of ubiquitination affected by USP10. Finally, the previous study showed that the organoids are a robust preclinical model [47]. Thus, in our further study, the organoids will be used to explore the effect of USP10 on the chemotherapy sensitivity of PDAC to GEM.

In summary, our study showed that USP10 was a tumor promoter and explored the underlying mechanism of USP10 in PDAC. USP10 was confirmed to be a new deubiquitinase of PLK1 that could increase the protein stability of PLK1. Moreover, USP10 suppression enhanced sensitivity to GEM chemotherapy by decreasing PLK1-mediated autophagy in vivo and in vitro. Thus, this study revealed that USP10 was a regulatory factor for GEM chemotherapy sensitivity and an attractive target for PDAC therapy.

## Supplementary information

Supplementary Figure 1

Supplementary Figure 2

Supplementary Figure 3

Supplementary Figure 4

Supplementary Figure 5

Supplementary Figure 6

Supplementary Figure 7

Supplementary Figure 8

Supplementary Figure 9

Supplementary Figure 10

Supplementary Table 1

Supplementary Table 2

Supplementary Table 3

Supplementary Table 4

Supplementary Table 5

Supplementary Table 6

Supplementary Table 7

Supplementary figure and table legends

WB raw data

## Data Availability

Data available on request from the authors upon reasonable request.
