# Supplementary figures and images for: USP10 promotes the progression and attenuates gemcitabine chemotherapy sensitivity via stabilizing PLK1 in PDAC
Source: Cell Death Dis. 2025 Jun 14;16(1):449. doi: 10.1038/s41419-025-07757-z (PMC12167373; doi:10.1038/s41419-025-07757-z)

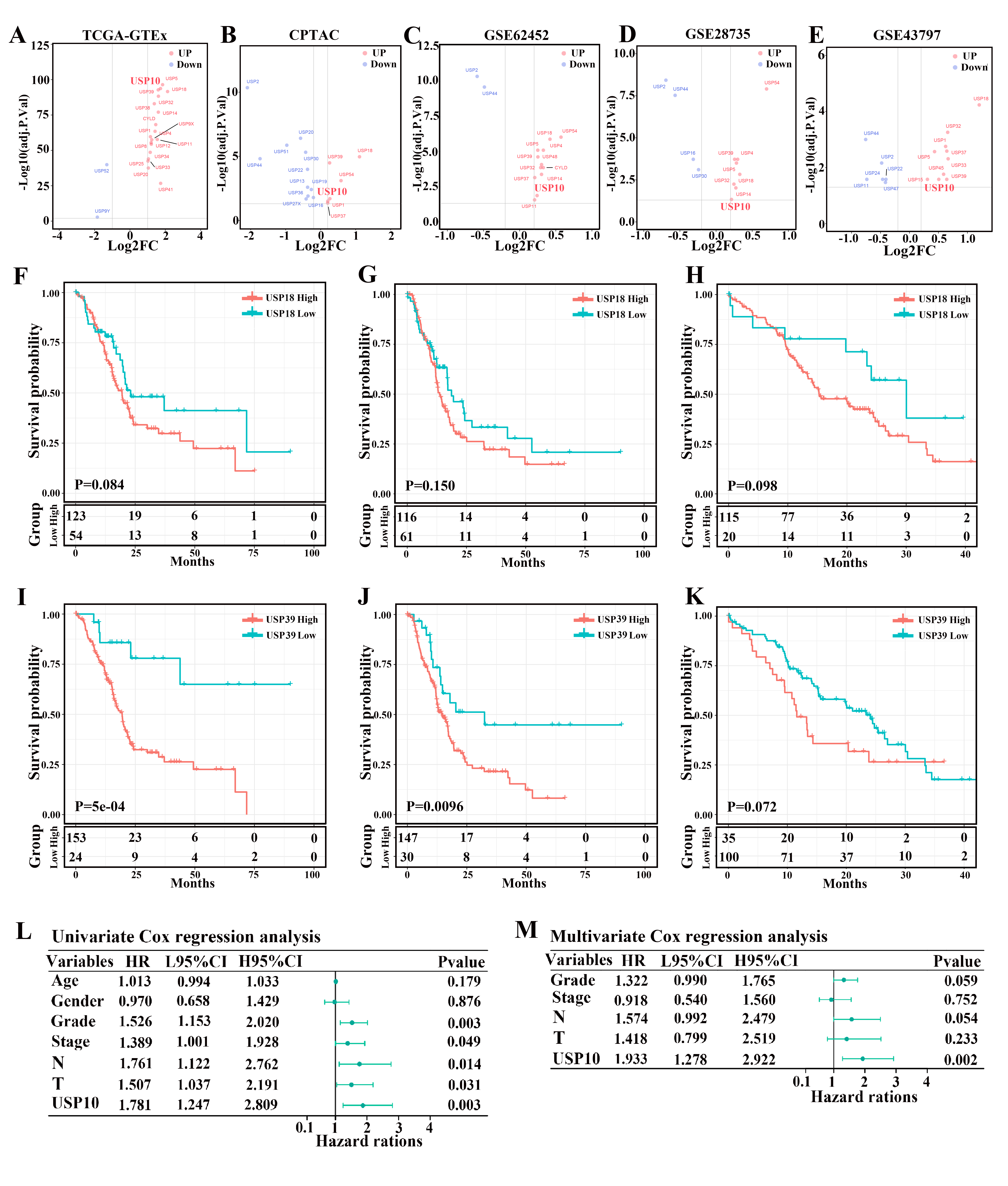

Supplement: Supplementary file 1 — Supplementary Figure 1 [file 41419_2025_7757_MOESM1_ESM.tif]

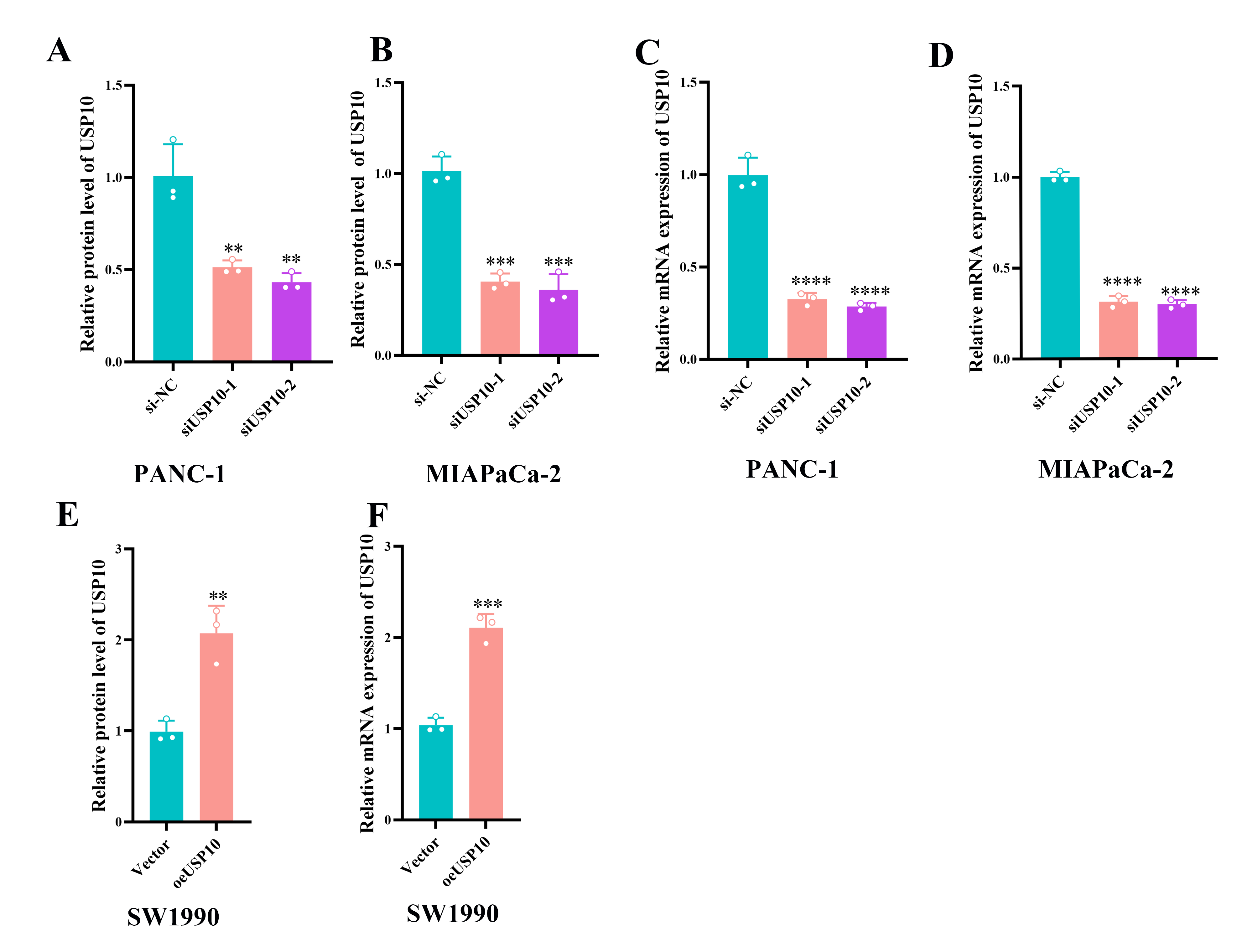

Supplement: Supplementary file 2 — Supplementary Figure 2 [file 41419_2025_7757_MOESM2_ESM.tif]

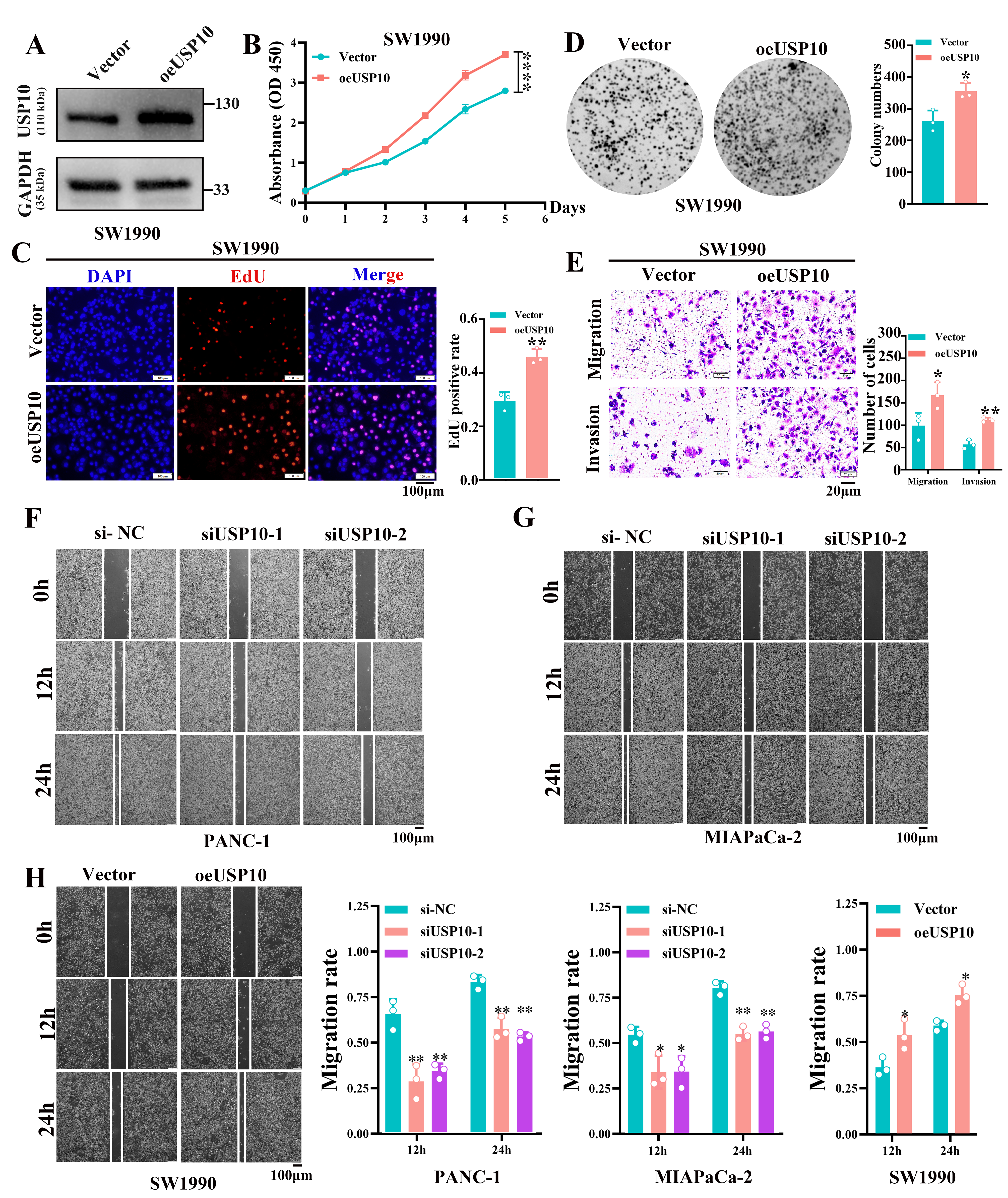

Supplement: Supplementary file 3 — Supplementary Figure 3 [file 41419_2025_7757_MOESM3_ESM.tif]

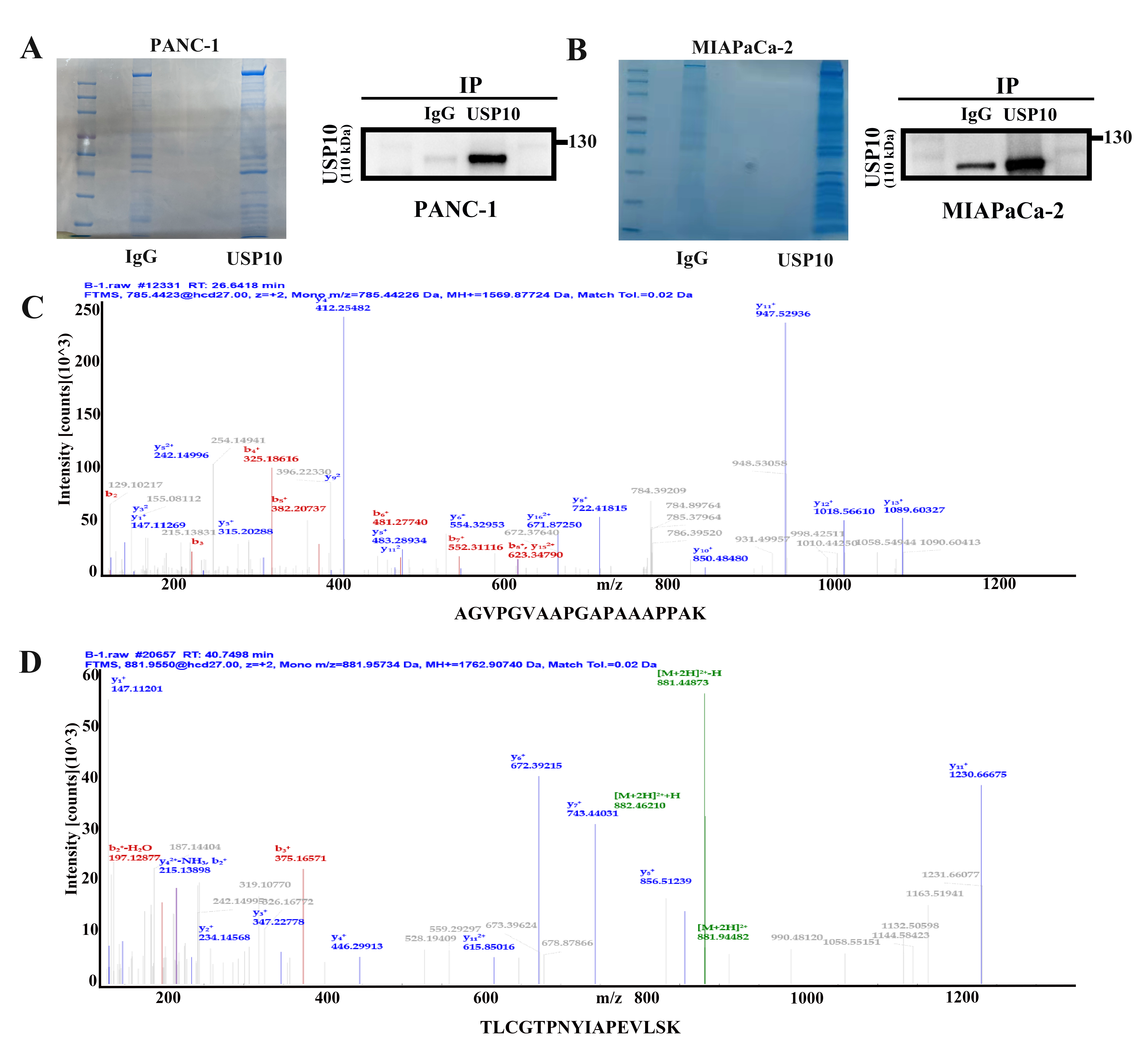

Supplement: Supplementary file 4 — Supplementary Figure 4 [file 41419_2025_7757_MOESM4_ESM.tif]

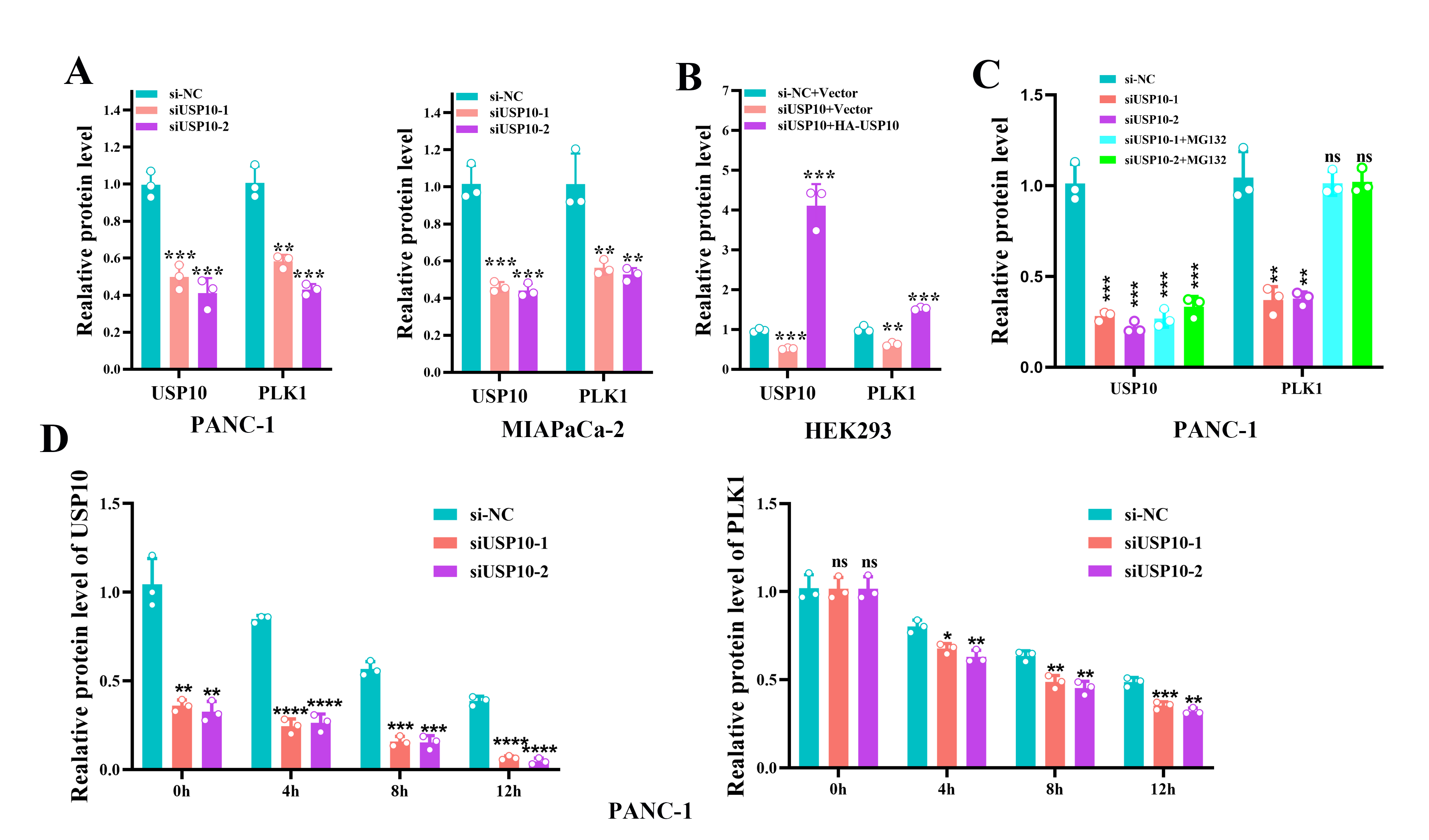

Supplement: Supplementary file 5 — Supplementary Figure 5 [file 41419_2025_7757_MOESM5_ESM.tif]

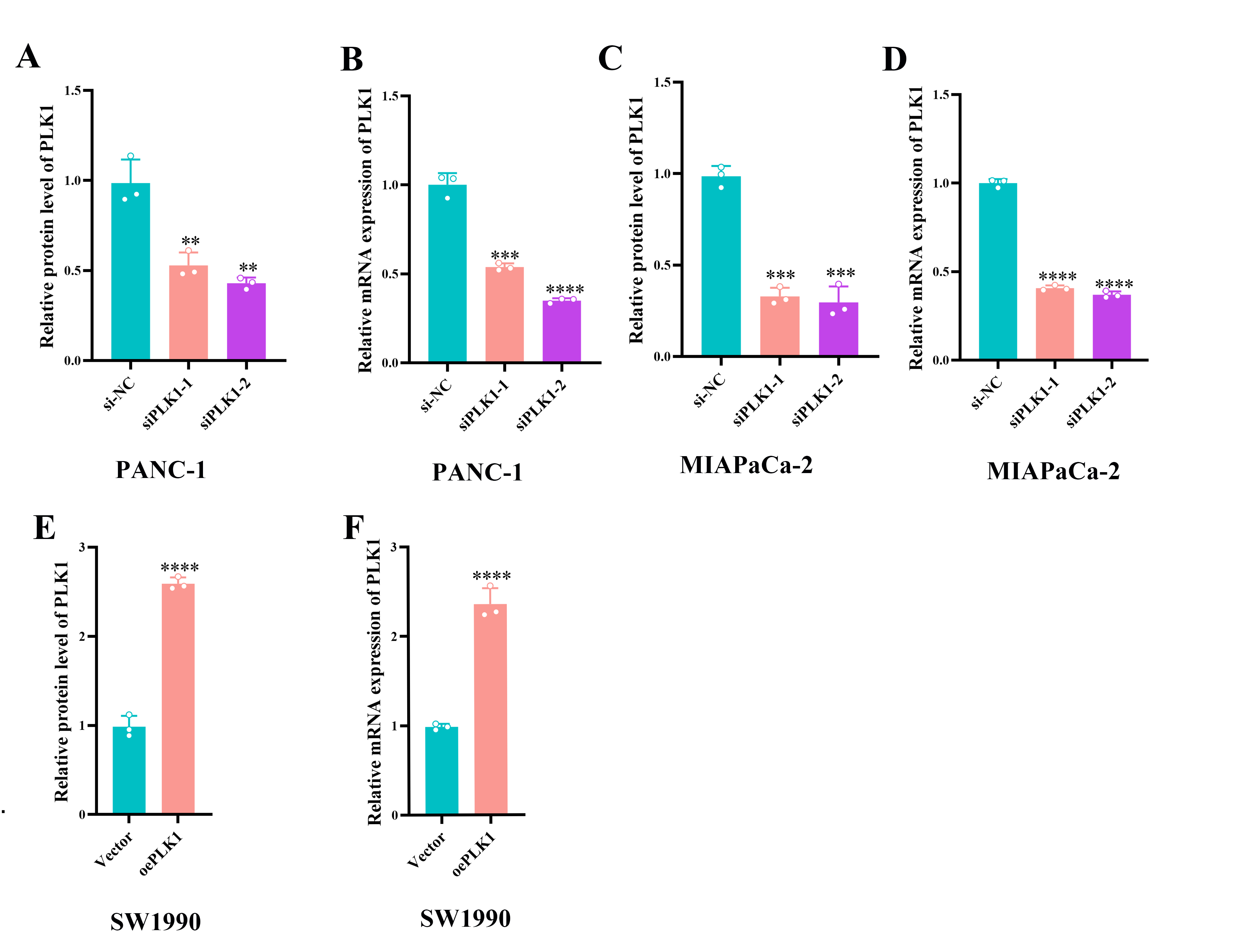

Supplement: Supplementary file 6 — Supplementary Figure 6 [file 41419_2025_7757_MOESM6_ESM.tif]

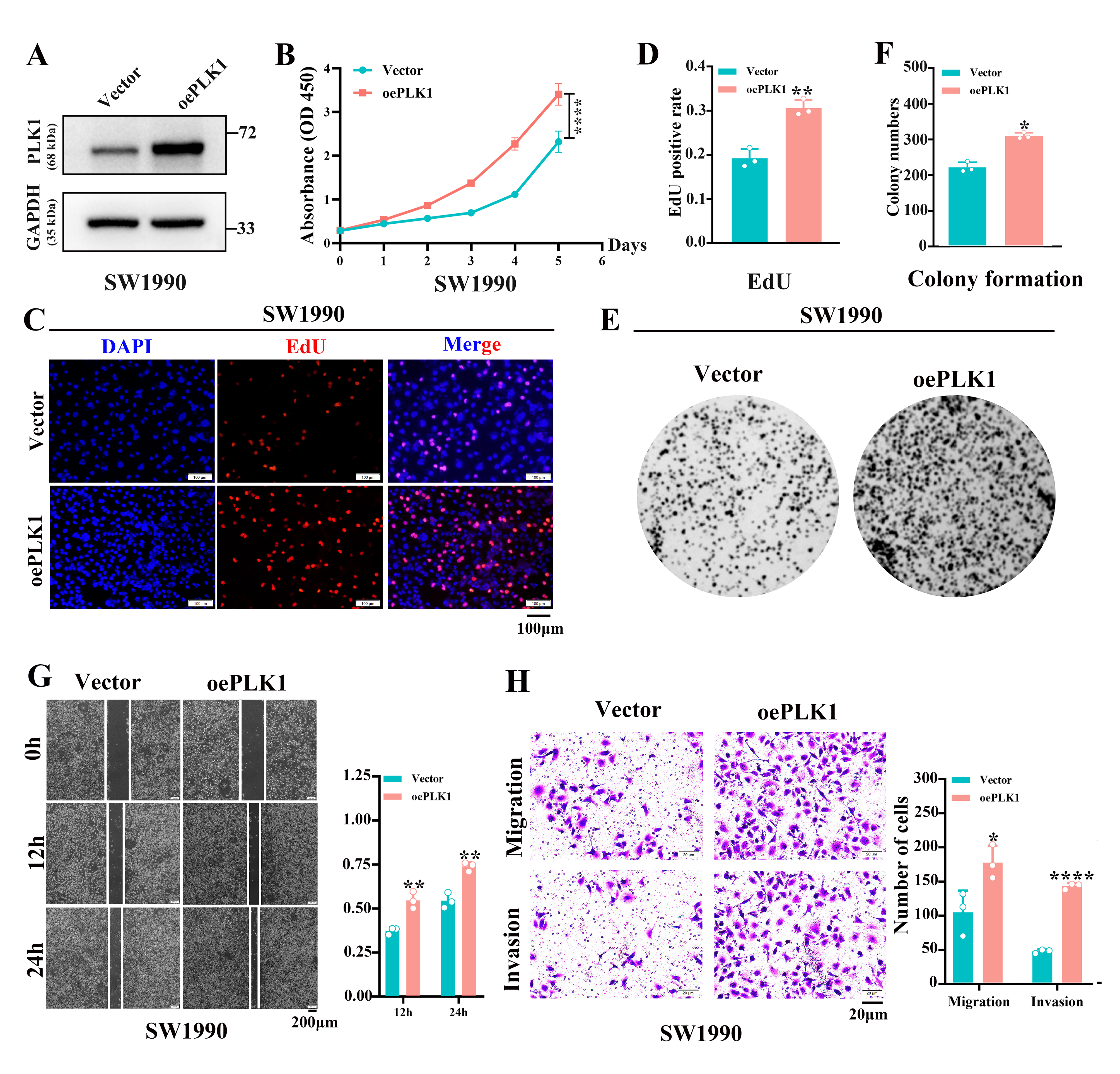

Supplement: Supplementary file 7 — Supplementary Figure 7 [file 41419_2025_7757_MOESM7_ESM.tif]

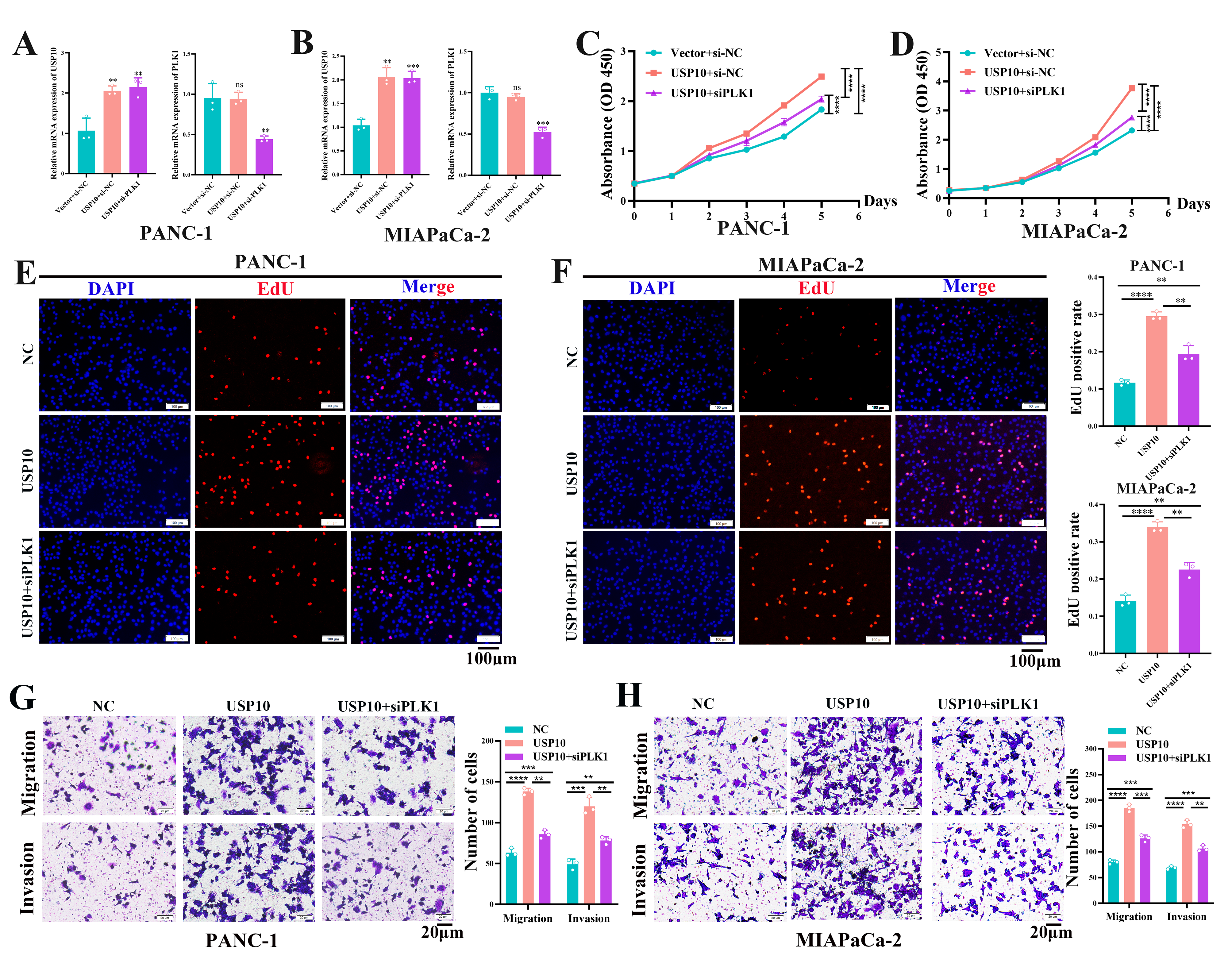

Supplement: Supplementary file 8 — Supplementary Figure 8 [file 41419_2025_7757_MOESM8_ESM.tif]

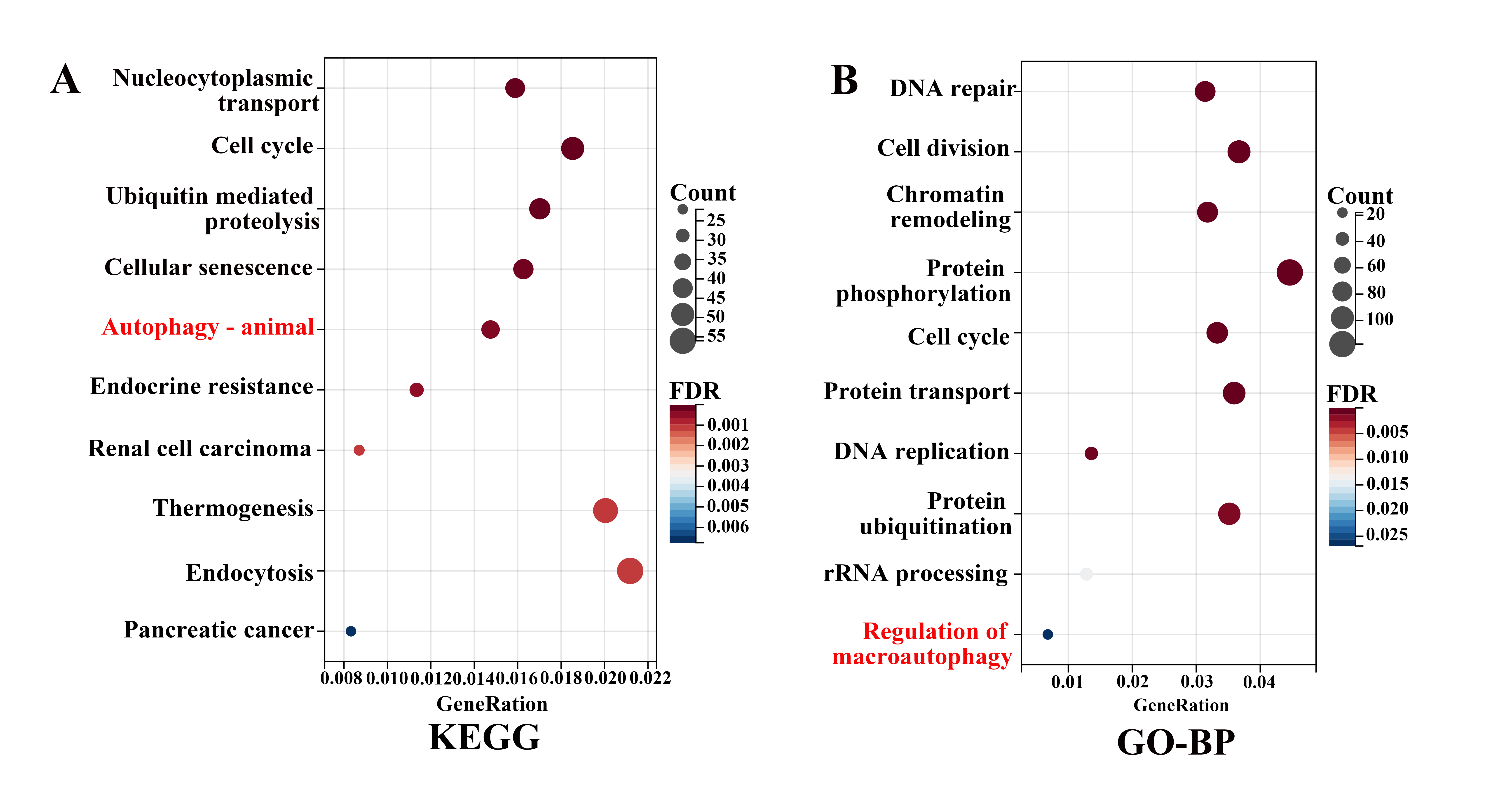

Supplement: Supplementary file 9 — Supplementary Figure 9 [file 41419_2025_7757_MOESM9_ESM.tif]

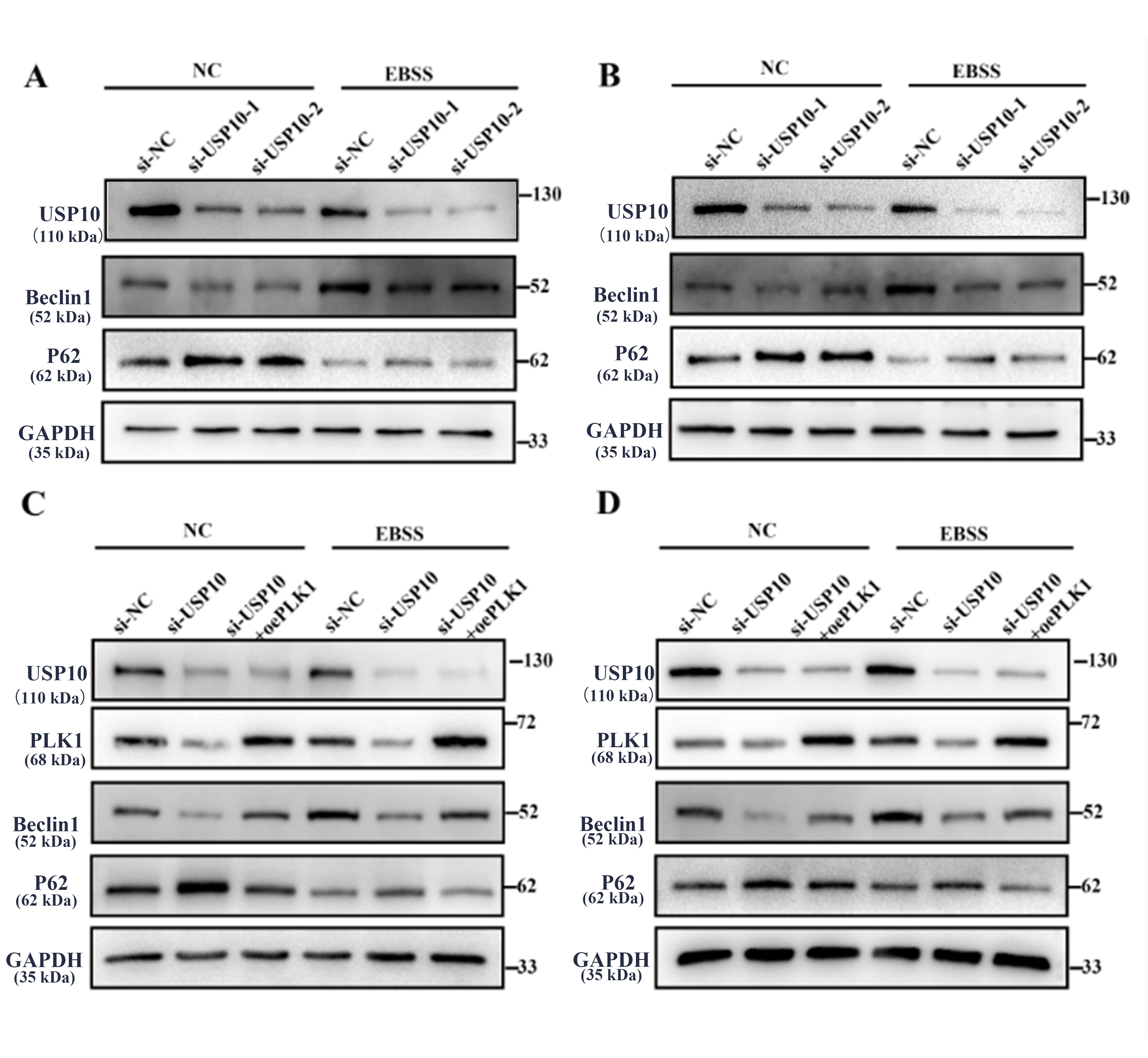

Supplement: Supplementary file 10 — Supplementary Figure 10 [file 41419_2025_7757_MOESM10_ESM.tif]

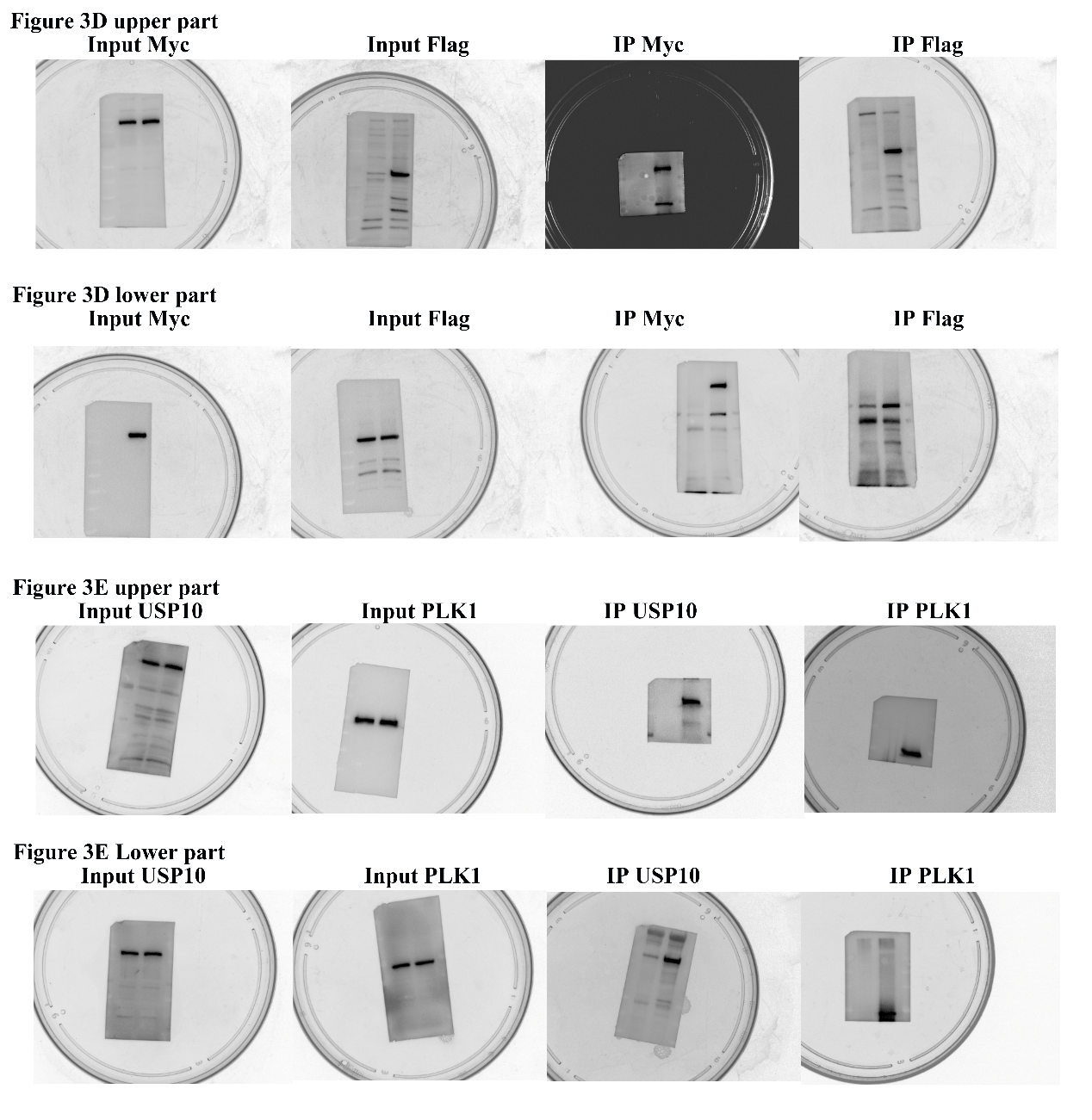


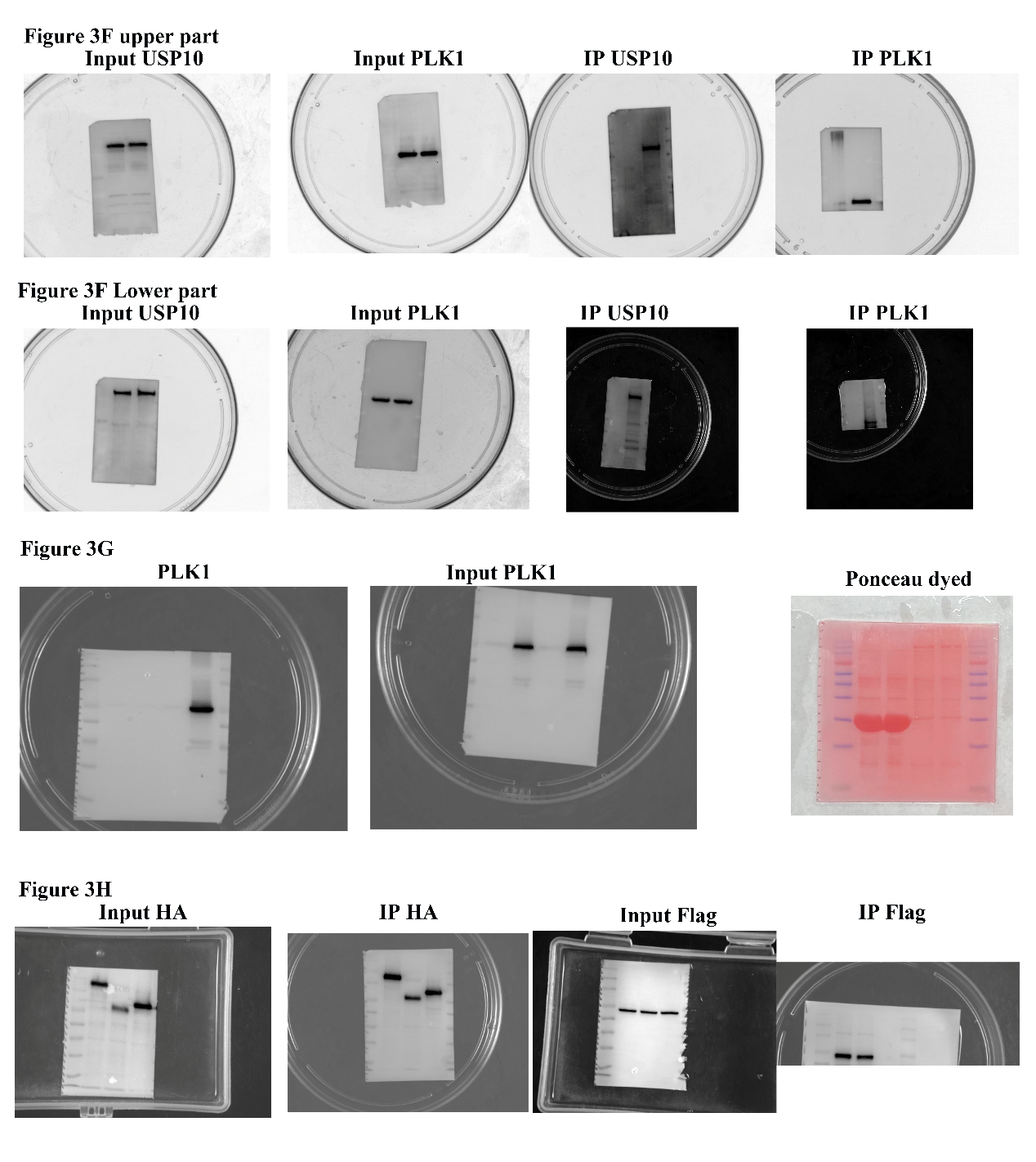


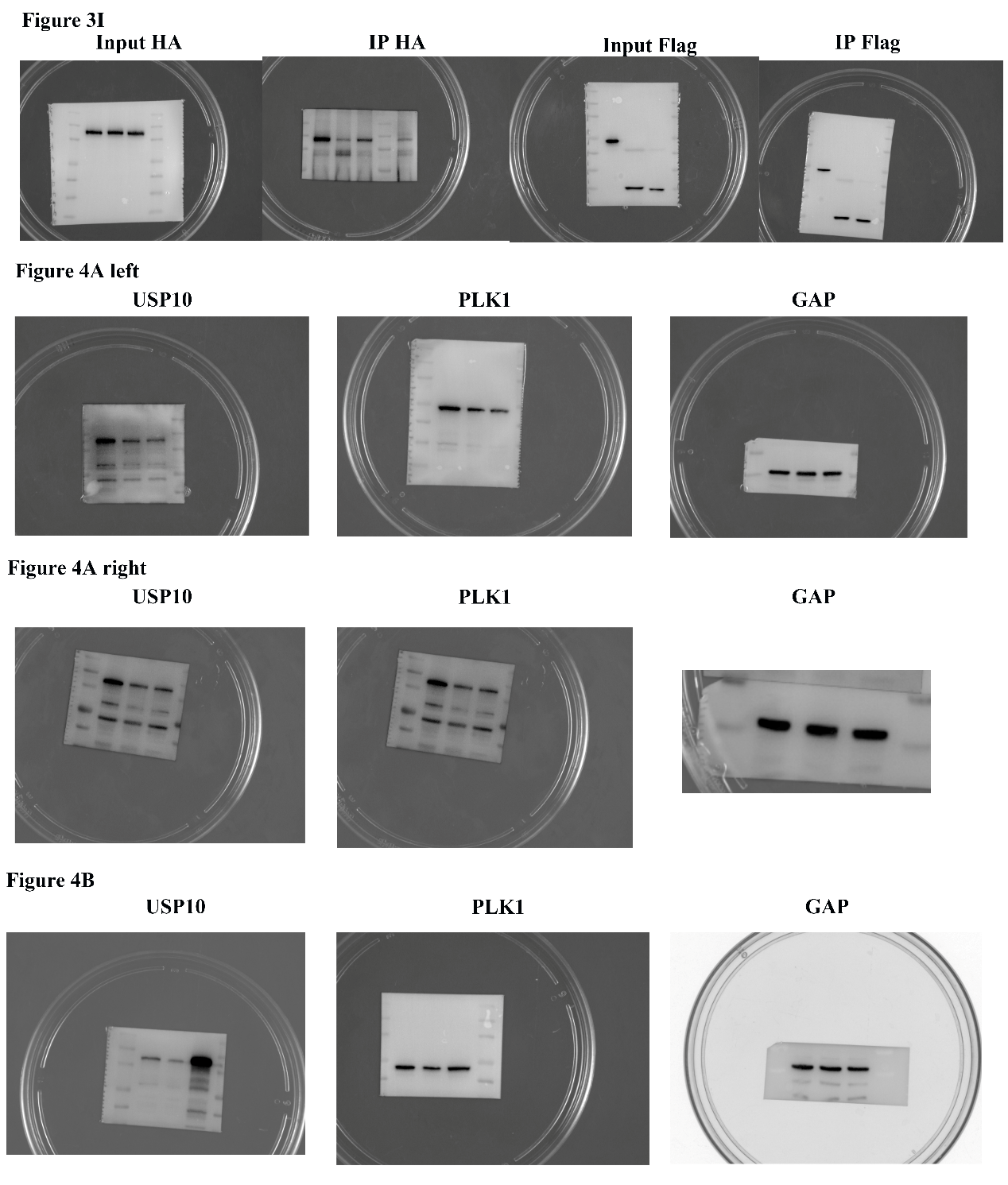


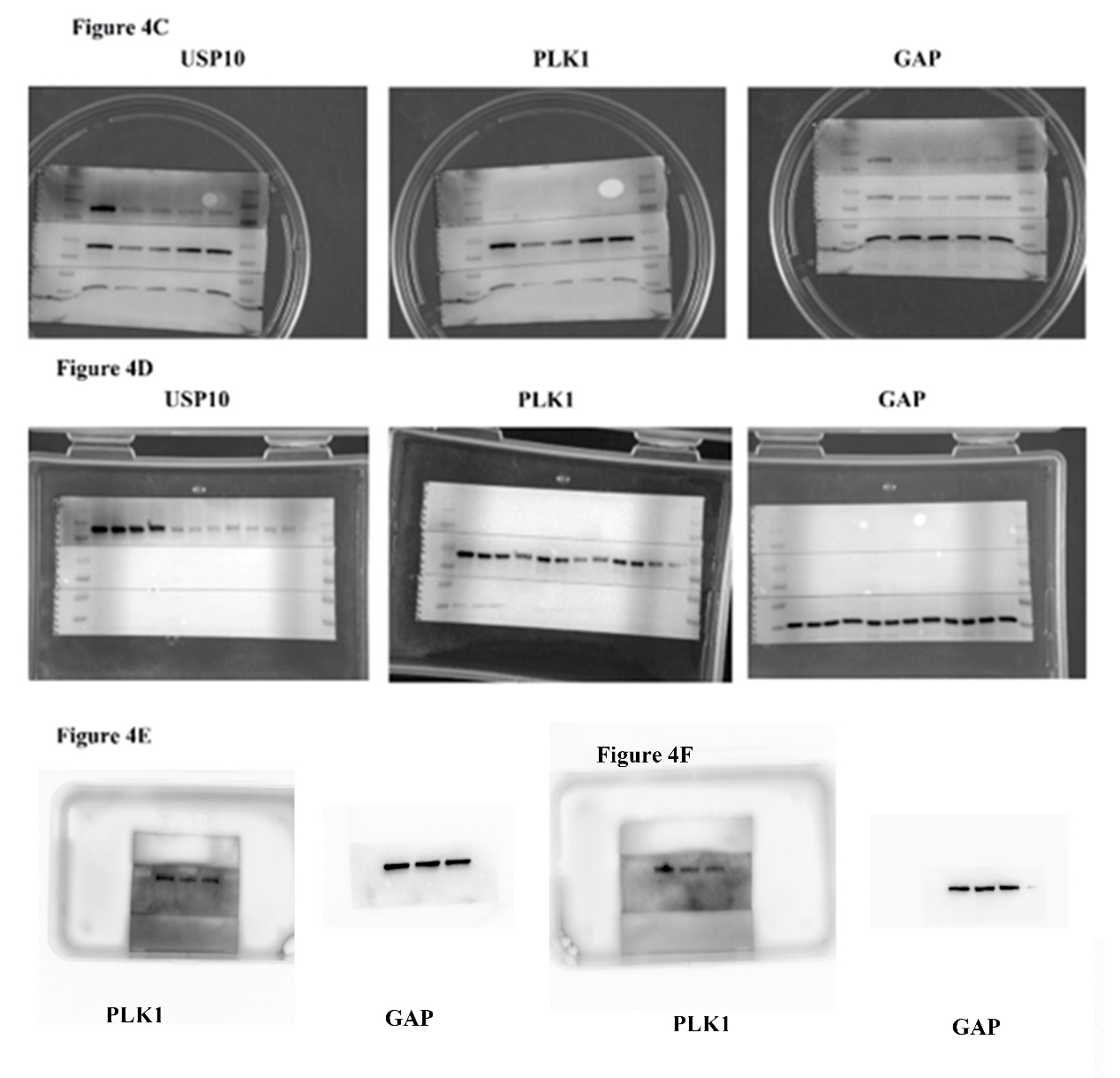


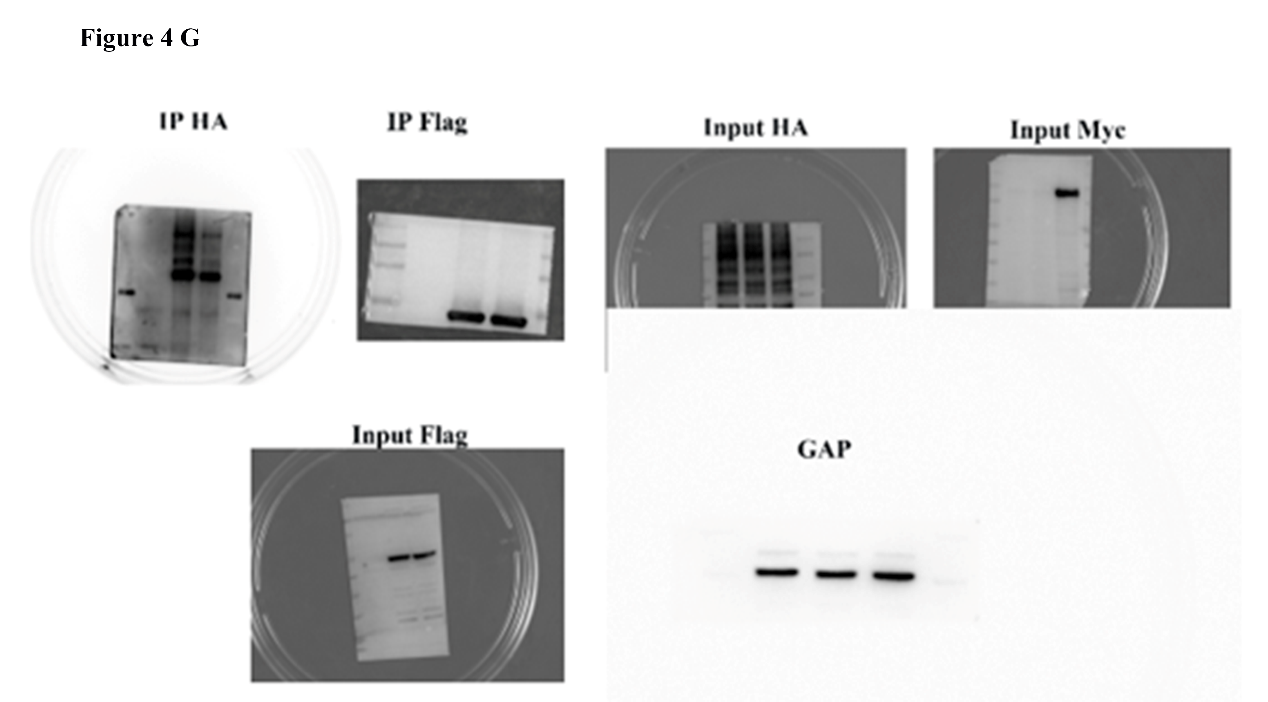


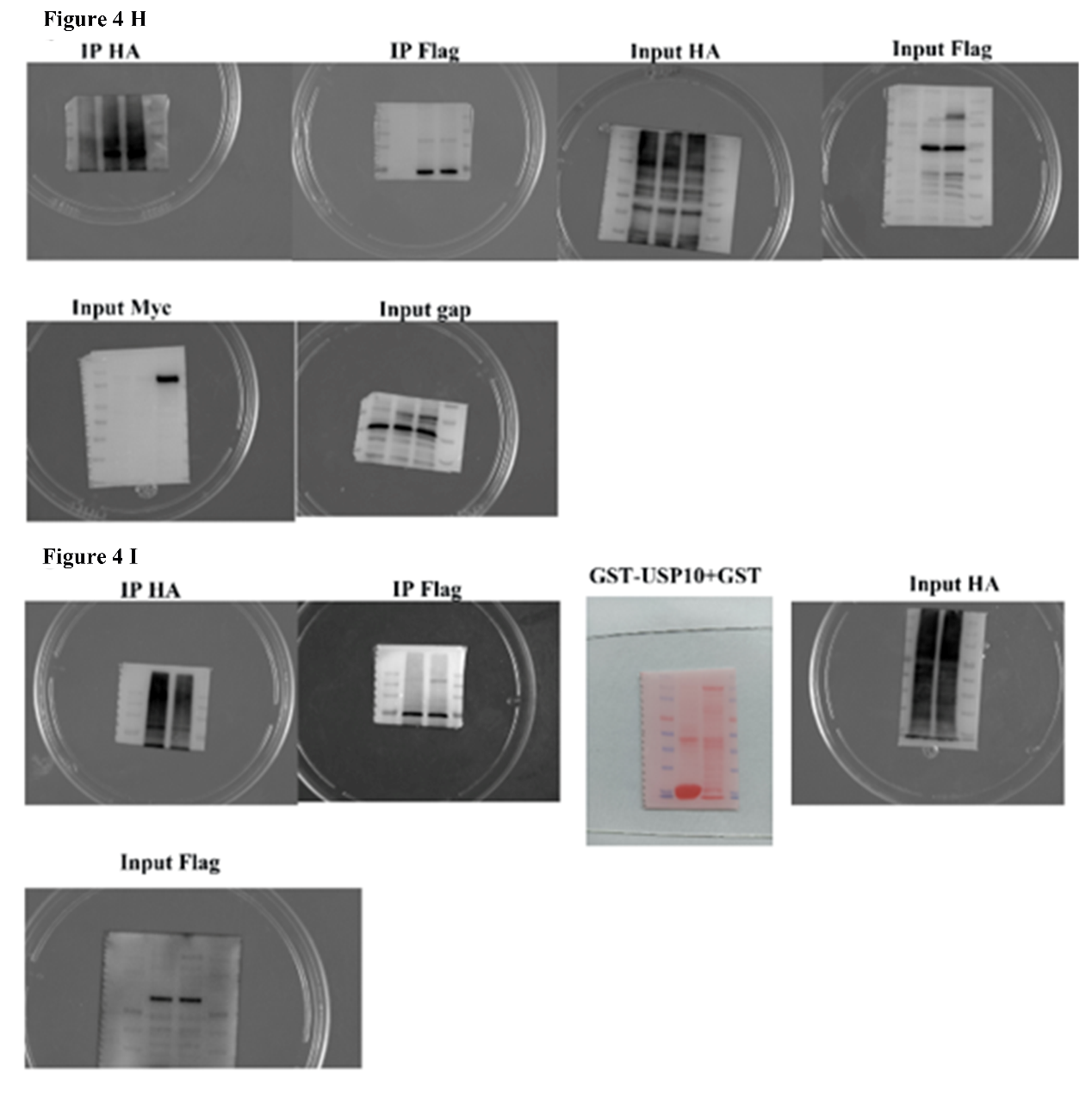


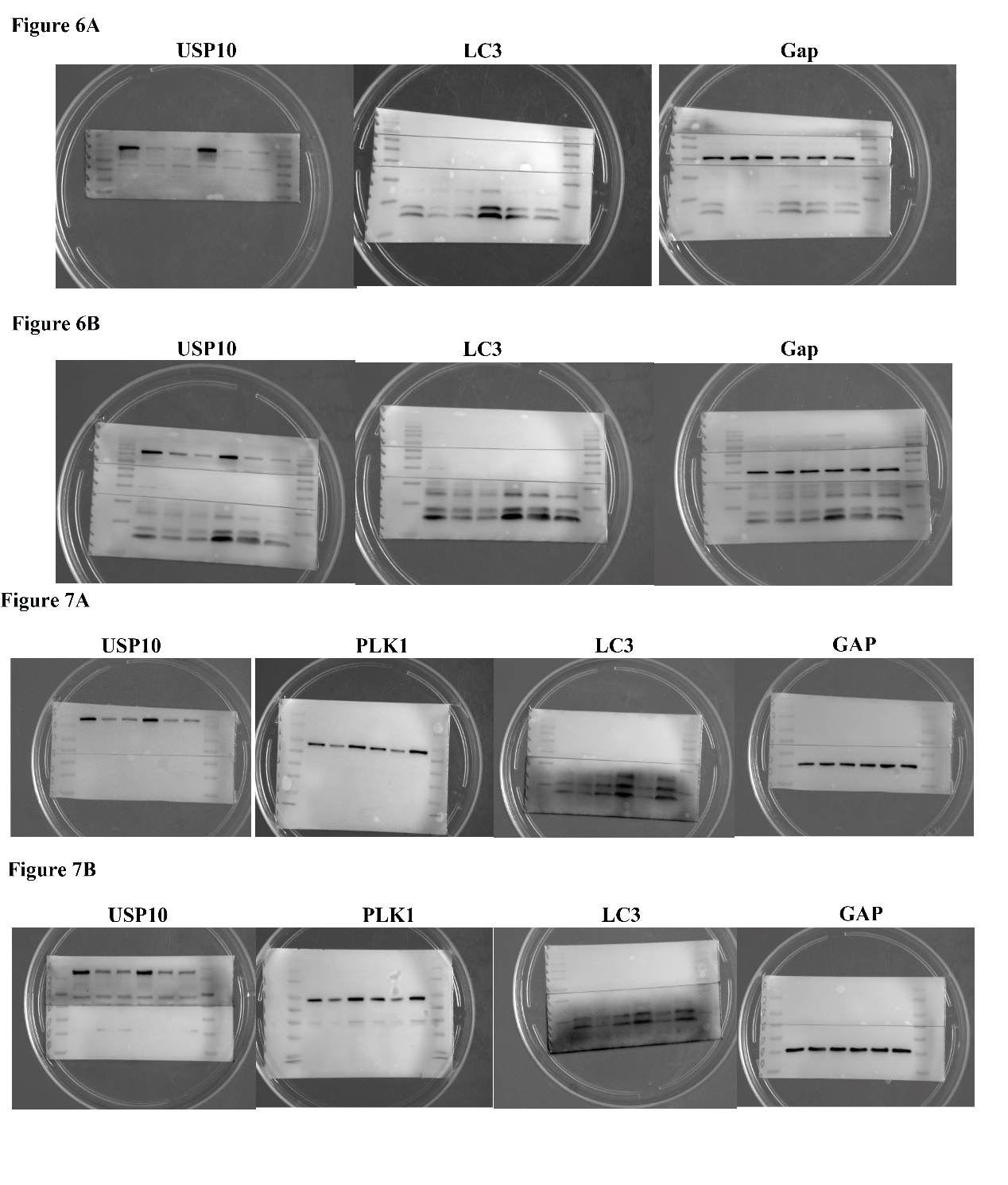


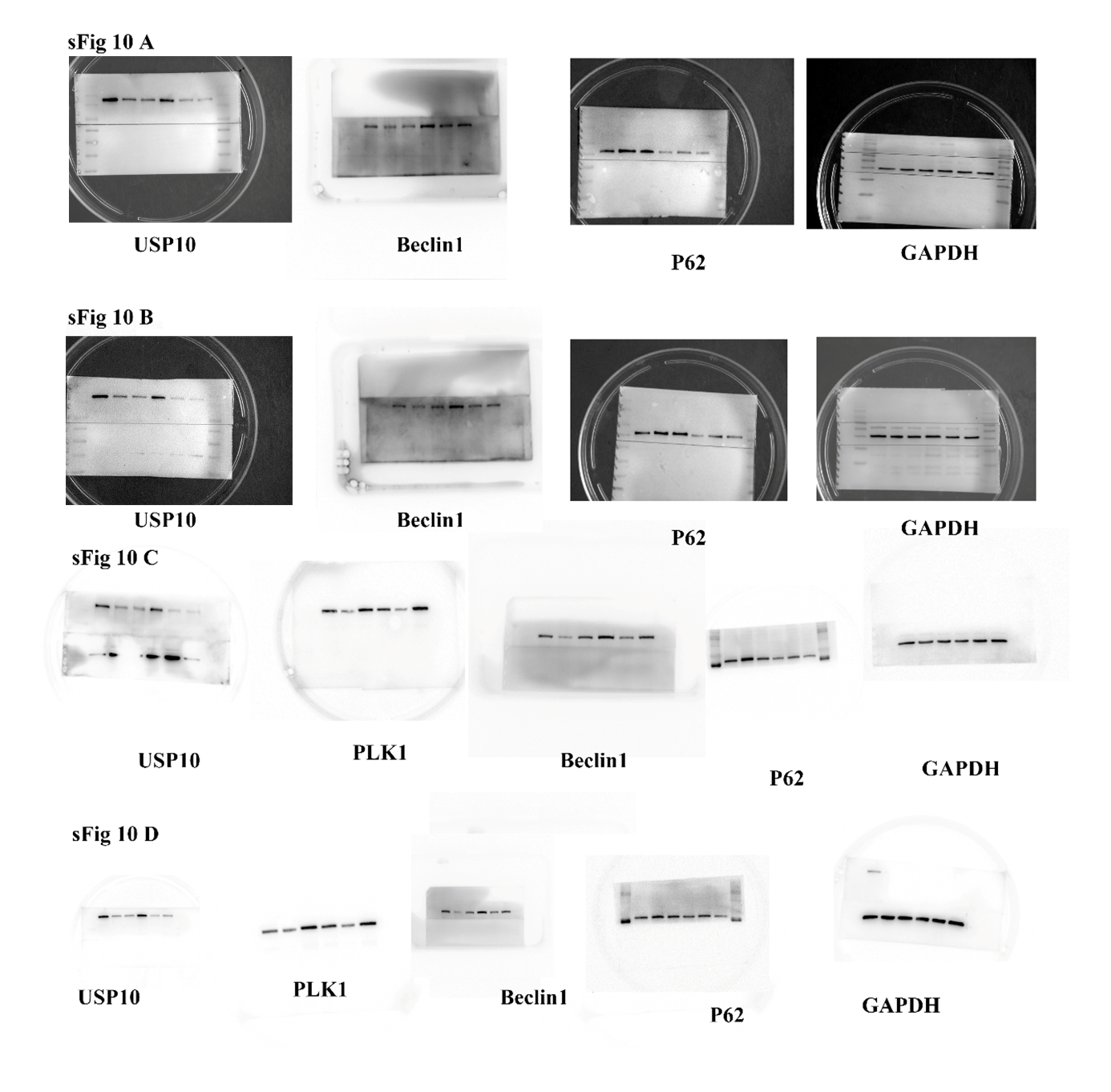

Supplement: Supplementary file 19 — WB raw data [file 41419_2025_7757_MOESM19_ESM.docx]
