# Supplementary material for: USP10 promotes the progression and attenuates gemcitabine chemotherapy sensitivity via stabilizing PLK1 in PDAC
Source: Cell Death Dis. 2025 Jun 14;16(1):449. doi: 10.1038/s41419-025-07757-z (PMC12167373; doi:10.1038/s41419-025-07757-z)
Supplement: Supplementary file 11 — Supplementary Table 1 [file 41419_2025_7757_MOESM11_ESM.docx]

**Supplementary table 1. The primer sequences in this study**

| Primer name | Forward | Reverse |
| --- | --- | --- |
| USP10 | CGGCTCTTTGCAGTGGTCTA | GTGCGTTCAGCAGTTGGTTT |
| PLK1 | TGACTCAACACGCCTCATCC | GCTCGCTCATGTAATTGCGG |
| GAPDH | GGTGGTCTCCTCTGACTTCAACA | GTTGCTGTAGCCAAATTCGTTGT |
