# Supplementary material for: USP10 promotes the progression and attenuates gemcitabine chemotherapy sensitivity via stabilizing PLK1 in PDAC
Source: Cell Death Dis. 2025 Jun 14;16(1):449. doi: 10.1038/s41419-025-07757-z (PMC12167373; doi:10.1038/s41419-025-07757-z)
Supplement: Supplementary file 12 — Supplementary Table 2 [file 41419_2025_7757_MOESM12_ESM.docx]

**Supplementary table 2. The siRNA sequences in this study**

| siRNA name | Sense | Antisense |
| --- | --- | --- |
| si-NC | UUCUCCGAACGUGUCACGUTT | ACGUGACACGUUCGGAGAATT |
| siUSP10-1 | CCAUAAAGAUUGCAGAGUUTT | AACUCUGCAAUCUUUAUGGTT |
| siUSP10-2 | CCACAUAUAUUUACAGACUTT | AGUCUGUAAAUAUAUGUGGTT |
| siPLK1-1 | CGAUACUACCUACGGCAAATT | UUUGCCGUAGGUAGUAUCGTT |
| siPLK1-2 | CGAGGUGCUGAGCAAGAAATT | UUUCUUGCUCAGCACCUCGCTT |
