# Supplementary material for: USP10 promotes the progression and attenuates gemcitabine chemotherapy sensitivity via stabilizing PLK1 in PDAC
Source: Cell Death Dis. 2025 Jun 14;16(1):449. doi: 10.1038/s41419-025-07757-z (PMC12167373; doi:10.1038/s41419-025-07757-z)
Supplement: Supplementary file 13 — Supplementary Table 3 [file 41419_2025_7757_MOESM13_ESM.docx]

**Supplementary Table 3. The detailed information of plasmids in this study**

| Symbol | Transcript ID | Amino acids sequence | Vector | Source |
| --- | --- | --- | --- | --- |
| Myc-USP10 | NM_005153 | Full length | pcDNA3.1 | GenScript Co., Ltd. |
| HA-USP10 F | NM_005153 | Full length | pcDNA3.1 | GenScript Co., Ltd. |
| HA-USP10 #1 | NM_005153 | 1-399 | pcDNA3.1 | GenScript Co., Ltd. |
| HA-USP10 #2 | NM_005153 | 206-798 | pcDNA3.1 | GenScript Co., Ltd. |
| GST-USP10 | NM_005153 | Full length | pGEX-4T-1 | GenScript Co., Ltd. |
| Flag-PLK1 F | NM_005030 | Full length | pcDNA3.1 | GenScript Co., Ltd. |
| Flag-PLK1 #1 | NM_005030 | 1-307 | pcDNA3.1 | GenScript Co., Ltd. |
| Flag-PLK1 #2 | NM_005030 | 307-603 | pcDNA3.1 | GenScript Co., Ltd. |
