# Supplementary material for: USP10 promotes the progression and attenuates gemcitabine chemotherapy sensitivity via stabilizing PLK1 in PDAC
Source: Cell Death Dis. 2025 Jun 14;16(1):449. doi: 10.1038/s41419-025-07757-z (PMC12167373; doi:10.1038/s41419-025-07757-z)
Supplement: Supplementary file 14 — Supplementary Table 4 [file 41419_2025_7757_MOESM14_ESM.docx]

**Supplementary table 4. The information of western blot antibodies in this study**

| **Name** | **Host** | **Dilution** | **Catalog** | **Socure** |
| --- | --- | --- | --- | --- |
| Anti-USP10 | Rabbit | 1:1000 | 19374-1-AP | Proteintech |
| Anti-GAPDH | Rabbit | 1:3000 | GB11002 | Servicebio |
| Anti-PLK1 | Rabbit | 1:1000 | 4513S | CST |
| Anti-Flag | Rabbit | 1:20000 | 20543-1-AP | Proteintech |
| Anti-Myc | Rabbit | 1:1000 | 16286-1-AP | Proteintech |
| Anti-HA | Rabbit | 1:1000 | 3724 | CST |
| Anti-GST | Mouse | 1:100 | SC138 | Santa Cruz |
| Anti-LC3B | Mouse | 1:100 | sc-398822 | Santa cruz |
