# Supplementary material for: USP10 promotes the progression and attenuates gemcitabine chemotherapy sensitivity via stabilizing PLK1 in PDAC
Source: Cell Death Dis. 2025 Jun 14;16(1):449. doi: 10.1038/s41419-025-07757-z (PMC12167373; doi:10.1038/s41419-025-07757-z)
Supplement: Supplementary file 15 — Supplementary Table 5 [file 41419_2025_7757_MOESM15_ESM.docx]

**Supplementary table 5. The components of NETN buffer.**

| **components** | **volume** | **concentration** |
| --- | --- | --- |
| 2M Tris-HCl（PH=8.0） | 8ml | 20mM |
| 5M NaCl | 16ml | 100mM |
| NP40 | 4ml | 0.5% |
| EDTA（PH=8.0） | 1.6ml | 1mM |
| ddH2O | 770.4ml | - |
