# Supplementary material for: USP10 promotes the progression and attenuates gemcitabine chemotherapy sensitivity via stabilizing PLK1 in PDAC
Source: Cell Death Dis. 2025 Jun 14;16(1):449. doi: 10.1038/s41419-025-07757-z (PMC12167373; doi:10.1038/s41419-025-07757-z)
Supplement: Supplementary file 16 — Supplementary Table 6 [file 41419_2025_7757_MOESM16_ESM.docx]

**Supplementary table 6. The components of deubiquitination buffer.**

| components | Volume/mass | concentration |
| --- | --- | --- |
| 1M Tris-HCl （PH=8.0） | 500μl | 50mmol/L |
| 5 M NaCl | 100μl | 50mmol/L |
| EDTA | 2.923mg | 1mmol/L |
| 1M DTT | 10μl | 1mmol/L |
| Glycerinum | 500μl | 5% |
| ddH2O | 8.9ml | - |
