# Supplementary material for: USP10 promotes the progression and attenuates gemcitabine chemotherapy sensitivity via stabilizing PLK1 in PDAC
Source: Cell Death Dis. 2025 Jun 14;16(1):449. doi: 10.1038/s41419-025-07757-z (PMC12167373; doi:10.1038/s41419-025-07757-z)
Supplement: Supplementary file 18 — Supplementary figure and table legends [file 41419_2025_7757_MOESM18_ESM.docx]

**sFig. 1 Screening process of USP family members (USPs) contributed to understanding the progression of PDAC.**

Volcano plots showing differentially expressed USPs in five public PDAC databases: TCGA-GTEx **(A)**, CPTAC **(B)**, GSE62452 **(C)**, GSE28735 **(D)**, and GSE43797 **(E)**. Correlation between USP18 expression levels and OS **(F)** and PFS **(G)** in TCGA database. **(H)** Correlation between USP18 expression and OS in the CPTAC cohort. Correlation between USP39 expression level and OS **(I)** and PFS **(J)** in the TCGA cohort. **(K)** Correlation between USP18 expression and OS in the CPTAC cohort. **(L-M)** Univariate and multivariate Cox regression analyses between USP10 and RFS in the TCGA database**.**

**sFig.2 The verification of USP10 interference efficiency**

**(A)** The knockdown efficiency of USP10 in PANC-1 validated by western blot. **(B)** The knockdown efficiency of USP10 in MIAPaCa-2 validated by western blot. **(C)** The knockdown efficiency of USP10 in PANC-1 validated by RT-qPCR. **(D)** The knockdown efficiency of USP10 in MIAPaCa-2 validated by RT-qPCR. **(E)** The overexpression efficiency of USP10 in SW1990 validated by western blot. **(F)** The overexpression efficiency of USP10 in SW1990 validated by RT-qPCR.

**sFig. 3** **Biological functions of USP10 in PDAC.**

**(A)** Validation of USP10 overexpression in SW1990 cells. The effect of USP10 overexpression on proliferation was detected using the CCK-8 assay **(B)**, EdU assay **(C)**, and colony formation assay **(D)**. **(E)** Transwell assays revealed that the migration and invasion were enhanced after USP10 overexpression in SW1990 cells. The migration ability after USP10 knockdown was detected with wound healing assays in PANC-1 **(F)** and MIAPaCa-2 **(G)** cells. **(H)** The migration ability after USP10 overexpression was detected through wound healing assays in SW1990 cells. Data are presented as mean ± sd. from 3 biologically independent samples. **P*<0.05, ***P* < 0.01, *****P* < 0.0001.

**sFig. 4 Quality control of IP-MS samples, and secondary spectra of PLK1 peptides.**

**(A-B)** Successful immunoprecipitation of USP10 was confirmed using western blot, and Coomassie brilliant blue staining showed that the abundance of USP10 was greater than that of IgG in PANC-1 and MIAPaCa-2 cells. **(C-D)** Secondary spectra revealed PLK1-unique peptides pulled down by USP10 in both PANC-1 and MIAPaCa-2 cells.

**sFig.5 The relative protein level of USP10 and PLK1.**

**(A)** The relative protein level of USP10 and PLK1 in Figure 4A. **(B)** The relative protein level of USP10 and PLK1 in Figure 4B. **(C)** The relative protein level of USP10 and PLK1 in Figure 4C. **(D)** The relative protein level of USP10 and PLK1 in Figure 4D. Data is presented as mean ± sd. from 3 biologically independent samples. **P*<0.05, ***P*<0.01, ****P* < 0.001, *****P* < 0.0001.

**sFig.6 The verification of PLK1 interference efficiency.**

**(A)** The knockdown efficiency of PLK1 in PANC-1 validated by western blot. **(B)** The knockdown efficiency of PLK1 in PANC-1 validated by RT-qPCR. **(C)** The knockdown efficiency of PLK1 in MIAPaCa-2 validated by western blot. **(D)** The knockdown efficiency of PLK1 in MIAPaCa-2 validated by RT-qPCR. **(E)** The overexpression efficiency of PLK1 in SW1990 validated by western blot. **(F)** The overexpression efficiency of PLK1 in SW1990 validated by RT-qPCR. Data is presented as mean ± sd. from 3 biologically independent samples. ***P* < 0.01, ****P* < 0.001, *****P* < 0.0001.

**sFig. 7 Biological functions of PLK1 in SW1990 cells.**

**(A)** Identification of PLK1 overexpression efficiency. The influence of USP10 overexpression on proliferation was detected through CCK-8 **(B)**, EdU **(C-D)**, and colony formation assays **(E-F)**. **(G)** The change in the migration ability after PLK1 overexpression was detected using a wound healing assay in SW1990 cells. **(H)** Transwell assay showed that migration and invasion were enhanced following PLK1 overexpression. Data is presented as mean ± sd. from 3 biologically independent samples. **P*<0.05, ***P* < 0.01, *****P* < 0.0001.

**sFig.8 The effect of USP10 overexpression on malignant biological behavior of PDAC cells can be partially recused by PLK1 knockdown.**

**(A)**The validation of interference efficiency of USP10 and PLK1 in PANC-1 cells. **(B)** The validation of interference efficiency of USP10 and PLK1 in MIAPaCa-2 cells. The proliferation ability was detected by CCK8 assays in PANC-1 **(C)** and MIAPaCa-2 **(D)** cells. The proliferation ability was detected by EdU assays in PANC-1 **(E)** and MIAPaCa-2 **(F)** cells. The migration and invasion abilities were detected by Transwell assays in PANC-1 **(G)** and MIAPaCa-2 **(H)** cells. Data is presented as mean ± sd. from 3 biologically independent samples. ***P*<0.01, ****P* < 0.001, *****P* < 0.0001.

**sFig. 9 Enrichment analysis of USP10-related genes.**

1. KEGG analysis of USP10-related genes. (B) GO-BP analysis of USP10-related genes.

**sFig.10 The change of p62 and Beclin1 protein level in different treated groups.**

**(A-B)**. The si-NC, si-USP10-1, si-USP10-2 were transfected into PDAC cells as required. The EBSS medium was used to activate autophagy. The p62 and Beclin1 were detected. **(C-D)**. The si-NC, si-USP10, and the overexpression plasmid of PLK1 were transfected into PDAC cells as required. The EBSS medium was used to activate autophagy. The p62 and Beclin1 were detected.

**Supplementary table 1**：The primer sequences in this study.

**Supplementary table 2：**The siRNA sequences in this study.

**Supplementary table 3:** The detailed information of plasmids in this study.

**Supplementary table 4:** The information of western blot antibodies in this study.

**Supplementary table 5:** The components of NETN buffer.

**Supplementary table 6:** The components of deubiquitination buffer.

**Supplementary table 7:** USP10-related genes.
